# [^225^Ac]Ac-iPSMA Radiopharmaceuticals for Targeted Alpha Therapy: Design, Synthesis, and Preclinical Evaluation

**DOI:** 10.3390/ijms27177785

**Published:** 2026-08-31

**Authors:** Daniel García-Arce, Myrna Luna-Gutiérrez, Pedro Cruz-Nova, Abraham Vidal-Limon, Nallely Jiménez-Mancilla, Liliana Aranda-Lara, Erika Azorín-Vega, Enrique Morales-Avila, Paola Rodríguez-López, Clara Santos-Cuevas, Guillermina Ferro-Flores, Blanca Ocampo-García

**Affiliations:** 1 Department of Radioactive Materials, Instituto Nacional de Investigaciones Nucleares, Ocoyoacac 52750, Mexico; danielgarcia.a1110@gmail.com (D.G.-A.); myrna.luna@inin.gob.mx (M.L.-G.); nova.pedro.servicios@inin.gob.mx (P.C.-N.); erica.azorin@inin.gob.mx (E.A.-V.); karenpaolarl12@gmail.com (P.R.-L.); clara.cuevas@inin.gob.mx (C.S.-C.); 2 Faculty of Chemistry, Universidad Autónoma del Estado de México, Toluca 50180, Mexico; emoralesav@uaemex.mx; 3 Red de Estudios Moleculares Avanzados, Instituto de Ecología A.C. (INECOL), El Haya, Xalapa 91073, Mexico; abraham.vidal@inecol.mx; 4 IxM SECIHTI-ININ, Department of Radioactive Materials, Instituto Nacional de Investigaciones Nucleares, Ocoyoacac 52750, Mexico; nallely.jimenez@inin.gob.mx; 5 Faculty of Medicine, Universidad Autónoma del Estado de México, Toluca 50180, Mexico; larandal@uaemex.mx

**Keywords:** Actinium-225, PSMA, HYNIC-iPSMA, targeted alpha therapy, monomeric, dimeric radiopharmaceutical, DOTA, MACROPA, combination therapy

## Abstract

Prostate-specific membrane antigen (PSMA) is a validated theranostic target with clinical relevance extending beyond prostate cancer to PSMA-expressing neovasculature in solid tumors. Despite the success of ^177^Lu-PSMA therapy, a significant proportion of patients experience disease recurrence or partial responses, motivating the development of targeted alpha therapy (TAT) approaches. In this study, novel monomeric and dimeric ^225^Ac-labeled PSMA inhibitors, [^225^Ac]Ac-DOTA-iPSMA ([^225^Ac]Ac-D-iPSMA) and [^225^Ac]Ac-MACROPA-iPSMA ([^225^Ac]Ac-M-iPSMA), were designed through receptor-based computational screening, synthesized, radiolabeled, and evaluated in vitro and in vivo. Radiolabeling achieved high radiochemical purities (>96%), and saturation binding assays using lanthanum(III) surrogates confirmed cooperative nanomolar PSMA affinity. The binding affinities were Kd = 8.28 ± 0.51 nM (La-D-iPSMA) and Kd = 16.76 ± 2.51 nM (La-M-iPSMA). PSMA-specific uptake was validated in 4T1 breast cancer and HCT116 colorectal cancer cells. D-iPSMA induced superior late apoptosis (41.74% at 2 Gy), associated with reductions in GSK-3 α/β (Glycogen Synthase Kinase-3) and WNK1 (With No Lysine Kinase 1) phosphorylation, β-catenin downregulation, and dose-dependent DNA double-strand breaks (72.08% γ-H2AX [γ-phosphorylated histone]-positive cells at 6 Gy). [^225^Ac]Ac-M-iPSMA biodistribution in 4T1 tumor-bearing BALB/c mice revealed rapid blood clearance, a tumor-to-kidney dose ratio of 54:1, and a high tumor-absorbed dose. A combination of [^177^Lu]Lu-D-iPSMA with [^225^Ac]Ac-M-iPSMA maximized reactive oxygen species generation. These findings support both conjugates as promising TAT candidates for PSMA-expressing tumors.

## 1. Introduction

Prostate-specific membrane antigen (PSMA) has become one of the most promising targets for radiopharmaceutical development, particularly in prostate cancer, and is also expressed in the neovasculature of solid tumors [1,2,3]. The development of PSMA-targeted beta-emitter lutetium-177 (^177^Lu) radiopharmaceuticals has shown remarkable clinical efficacy in patients with metastatic castration-resistant prostate cancer (mCRPC) [4,5,6,7]. In clinical trials, patients with mCRPC treated with [^177^Lu]Lu-iPSMA achieved a median overall survival of 21.7 months with an overall response rate of 36.6%. Some patients experienced complete clearance of all lesions, while others exhibited partial responses, defined as at least 30% reduction in lesion size [6].

Despite the therapeutic efficacy of [^177^Lu]Lu-PSMA inhibitors, a significant proportion of patients experience treatment recurrence or only partial responses. Recent evidence indicates that patients harboring mutations in Tumor Suppressor Genes (TSGs) such as TP53, PTEN, and RB1 can undergo neuroendocrine transdifferentiation, in which tumor cells acquire neuroendocrine characteristics associated with increased aggressiveness and treatment resistance [8]. Other abnormalities, such as mutations in CDK12, CCNE1, or FGFR1, may be associated with a lower treatment response [9].

Targeted alpha therapy (TAT) has emerged as a novel approach in nuclear medicine, offering the potential for highly localized cancer treatment with minimal damage to surrounding healthy tissue [10,11]. Among alpha-emitting radionuclides, actinium-225 (^225^Ac) exhibits favorable decay characteristics, including a 10 d half-life and the emission of 4 alpha particles per decay chain, delivering a cumulative energy of 28 MeV [12,13]. Emerging [^225^Ac]Ac-PSMA radiopharmaceuticals have demonstrated significant advances in mCRPC. Clinical data indicate that [^225^Ac]Ac-PSMA (including [^225^Ac]Ac-PSMA-617, [^225^Ac]Ac-PSMA-I&T, and [^225^Ac]Ac-J591) achieved a PSA50 (≥50% reduction in PSA levels) in 65% of cases, with better results in patients who had no prior treatment (82%) [14]. A different report showed benefits in about two-thirds of patients as a third-line treatment and also proved effective in managing prostate cancer as a second-line treatment [15]. Moreover, complete remissions were observed in 10% of patients treated with [^225^Ac]Ac-PSMA-617 [15,16]. Despite promising results, challenges remain in optimizing ^225^Ac radiopharmaceuticals, particularly regarding toxicity management and enhancement of therapeutic efficacy when used alone or in combination with ^177^Lu. Several studies have reported dose-limiting toxicities, including xerostomia and hematologic adverse events, which complicate clinical translation [14].

One of the main challenges in implementing ^225^Ac-TAT is the requirement for a bifunctional chelator that binds to the Ac^3+^ ion. PSMA-targeting ligands have been conjugated to 1,4,7,10-tetraazacyclododecane-1,4,7,10-tetraacetic acid (DOTA) to complex trivalent metal ions, such as ^225^Ac^3+^. While DOTA is considered the current standard chelator, its slow kinetics and requirement for elevated temperatures during radiolabeling limit its applicability with sensitive biomolecules [17]. Alternative chelators and bifunctional derivatives have been proposed, while comparative stability and biological behavior remain under investigation [18,19]. Thiele et al. described the 18-membered macrocyclic (MACROPA) and NCS-MACROPA as promising chelators for ^225^Ac, demonstrating rapid complexation at room temperature [20].

Dimeric radiopharmaceutical molecules have been shown to enhance target binding through increased avidity effects. Heterodimeric ^225^Ac-based radiopharmaceuticals have demonstrated enhanced tumor uptake and safer clinical profiles, dual targeting albumin and PSMA [21]. By incorporating two targeting moieties within a single molecular framework, dimeric constructs can achieve improved tumor uptake and retention compared to their monomeric counterparts [22].

In ^99m^Tc-PSMA SPECT (Single-Photon Emission Computed Tomography) imaging, HYNIC-iPSMA has been clinically validated as an effective molecule for improving the detection of prostate cancer lesions due to its high affinity for PSMA [23,24,25,26]. Moreover, integrating HYNIC-iPSMA can significantly enhance targeting for therapeutic applications, as demonstrated by [^177^Lu]Lu-iPSMA, thereby improving therapeutic outcomes in patients with mCRPC [6,27,28].

To improve the treatment of PSMA-positive cancers through ^225^Ac-endoradiotherapy, we hypothesized that HYNIC-iPSMA derivatives in both monomeric and dimeric configurations would retain the binding affinity when conjugated to either DOTA or MACROPA chelators for ^225^Ac^3+^. Based on these considerations, this study aims to design, synthesize, and evaluate the preclinical potential of [^225^Ac]Ac-MACROPA-iPSMA dimer ([^225^Ac]Ac-M-iPSMA) and [^225^Ac]Ac-DOTA-iPSMA monomer ([^225^Ac]Ac-D-iPSMA) as next-generation targeted alpha therapeutics for PSMA-expressing tumors.

## 2. Results and Discussion

### 2.1. Intermolecular Interactions Evaluated with Molecular Docking

**Figure 1:**
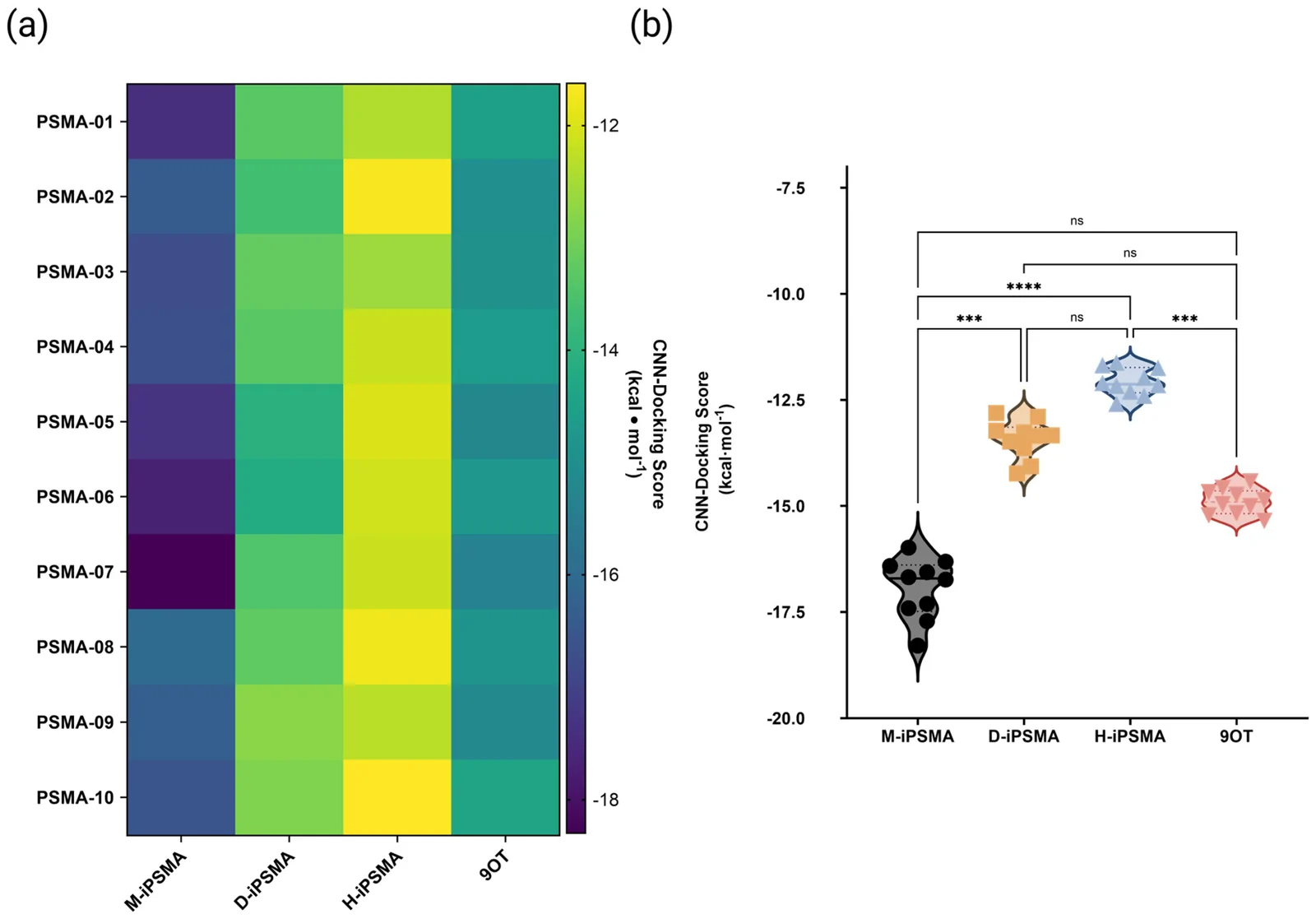
Receptor-based virtual screening of PSMA. Molecular docking evaluation of 3 different iPSMA vs. PSMA receptor structures. (**a**) Table of binding free energy per iPSMA. (**b**) Heatmap plot of binding free energy per iPSMA structure. M-iPSMA (MACROPA-iPSMA), D-iPSMA (DOTA-NCS-iPSMA), H-iPSMA (HYNIC-iPSMA). Statistical differences were determined by Kruskal-Wallis test followed by Dunn’s multiple comparisons test. *** *p* < 0.001, **** *p* < 0.0001; ns, not significant.

The ensemble docking approach provided insight into the affinity and binding modes of the evaluated ligands compared with endogenous and synthetic controls (Figure S1, Supplementary Materials). M-iPSMA docking within the PSMA active site (PDB: 5O5T) revealed a binding pattern consistent with reported motifs, with unique adaptations from its hybrid chelator–linker structure, supporting a multi-anchor binding model where conserved enzyme residues ensure recognition despite ligand structural differences (Figure 1).

An important interaction was observed with ARG534 (Table 1), located within the S1 hydrophobic pocket that defines the arginine patch (ARG463, ARG534, and ARG536). ARG534 plays a critical structural role in maintaining an invariant orientation through electrostatic coupling with the S1-bound chloride anion. For M-iPSMA, the terminal glutamic acid forms a direct hydrogen bond with the guanidinium group of ARG534, replacing the chloride-mediated contact reported in earlier studies. This substitution preserves the electrostatic landscape of the S1 pocket while reinforcing the anchoring of the conjugate.

ARG534, within the S1 arginine patch (ARG463, ARG534, ARG536), maintains an invariant orientation through electrostatic coupling with the S1-bound chloride anion. In M-iPSMA, the terminal glutamic acid forms a direct hydrogen bond with ARG534′s guanidinium group, replacing the chloride-mediated contact and preserving the S1 electrostatic landscape.

TYR700, at the S1′ pocket entrance, forms a hydrogen bond with the MACROPA macrocycle carbonyl and a predicted π–π stacking contact with the HYNIC linker pyridine ring in M-iPSMA, differing from the α-carboxylate H-bond reported by Nikfarjam et al. [29]. These interactions should be interpreted as computationally predicted binding features rather than direct experimental evidence of receptor conformational flexibility or enhanced complex stability. TRP541, a key hydrophobic anchor [30], shows a conserved predicted π–π stacking contact with the M-iPSMA naphthyl ring; therefore, the extended aromatic surface may contribute to ligand accommodation within the binding pocket, although this hypothesis requires experimental validation.

SER503, ILE512, TYR471, and TYR692 extend the hydrogen-bonding network along the substrate channel, complementing the dominant electrostatic (ARG463, ARG534, LYS610) and aromatic (PHE209, TRP541, TYR700) interactions. Together, these docking-derived contacts suggest a plausible multi-anchor binding mode for M-iPSMA, but they do not by themselves demonstrate experimentally confirmed high-affinity binding or complex stability.

**Figure 2:**
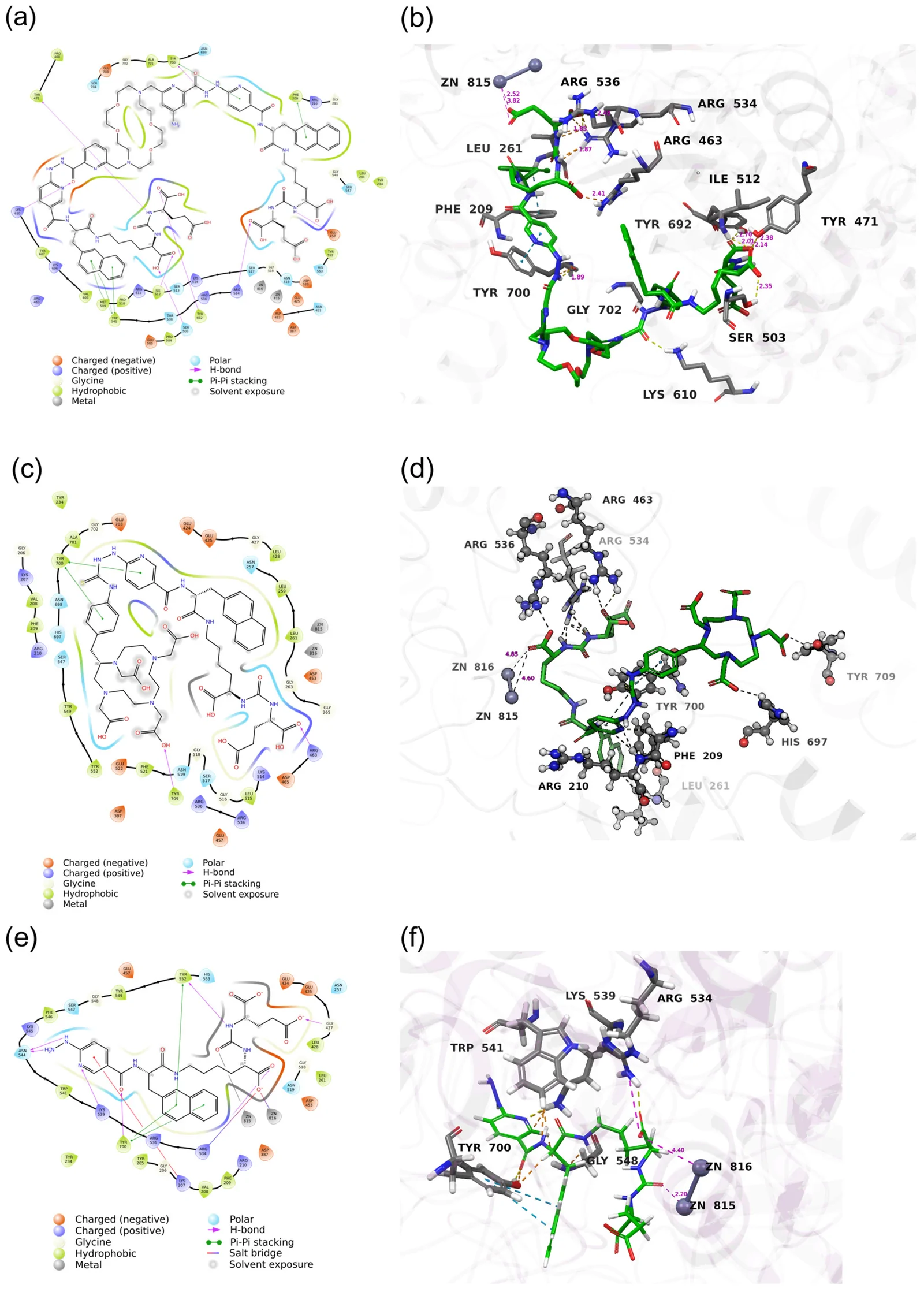
Molecular Docking Evaluation. (**a**,**b**) Two-dimensional and 3D ligand interaction diagrams for M-iPSMA at the PSMA binding site. (**c**,**d**) Two-dimensional and 3D diagrams for D-iPSMA; (**e**,**f**) for HYNIC-iPSMA. Distances between Zn^2+^ and critical carbonyl oxygens are highlighted. The protein appears as a ribbon; Zn^2+^ (purple) and ligand (green) are ball-and-stick.

The docking model suggests that the glutamate carboxylate is positioned near the catalytic Zn^2+^ center (Zn815), with metal–oxygen distances compatible with a potential metal-assisted interaction; however, this should be regarded as a computational prediction rather than experimental confirmation of zinc coordination. This interpretation is consistent with Figure 2a,b and Table 1.

D-iPSMA engages PHE209, ARG210, LEU261, ARG463, ARG534, ARG536, and TYR700 via π–π stacking, π–cation contacts, and hydrogen bonds, anchoring the glutamate–urea motif as in validated PSMA inhibitors. This multi-anchor stabilization ensures high-affinity binding despite the DOTA chelator.

HYNIC-iPSMA maintains the iPSMA recognition pattern: the HYNIC pyridine forms a π–cation interaction with LYS207; Glu COOH hydrogen-bonds GLY427 and salt-bridges ARG534; TYR552 and TYR700 contribute additional H-bonds and π-stacking. Coordination of Zn815 with the urea carbonyl and Zn816 with the ARG534-bound carboxylate indicates bimetallic stabilization of the binding motif (Figure 2e,f).

### 2.2. Dynamic Behavior of iPSMA Complexes

MD analyses were used to evaluate the persistence of predicted binding poses for D-iPSMA and M-iPSMA within the PSMA active site. The 500 ns RMSD (root-mean-square deviation) profiles (Figures S2–S4) showed stable plateaus after equilibration, supporting the computational stability of the simulated complexes under the selected modeling conditions, without implying direct experimental validation of complex stability.

The M-iPSMA–PSMA complex showed RMSF (Root Mean Square Fluctuation) values of 4–5 Å, compared with previously reported values greater than 10 Å [29]. Lower fluctuations indicate reduced mobility, possibly due to stabilizing contacts in the multidentate MACROPA and π-rich HYNIC-iPSMA surfaces. However, this is computational and not direct evidence of increased receptor rigidity or stabilization.

**Figure 3:**
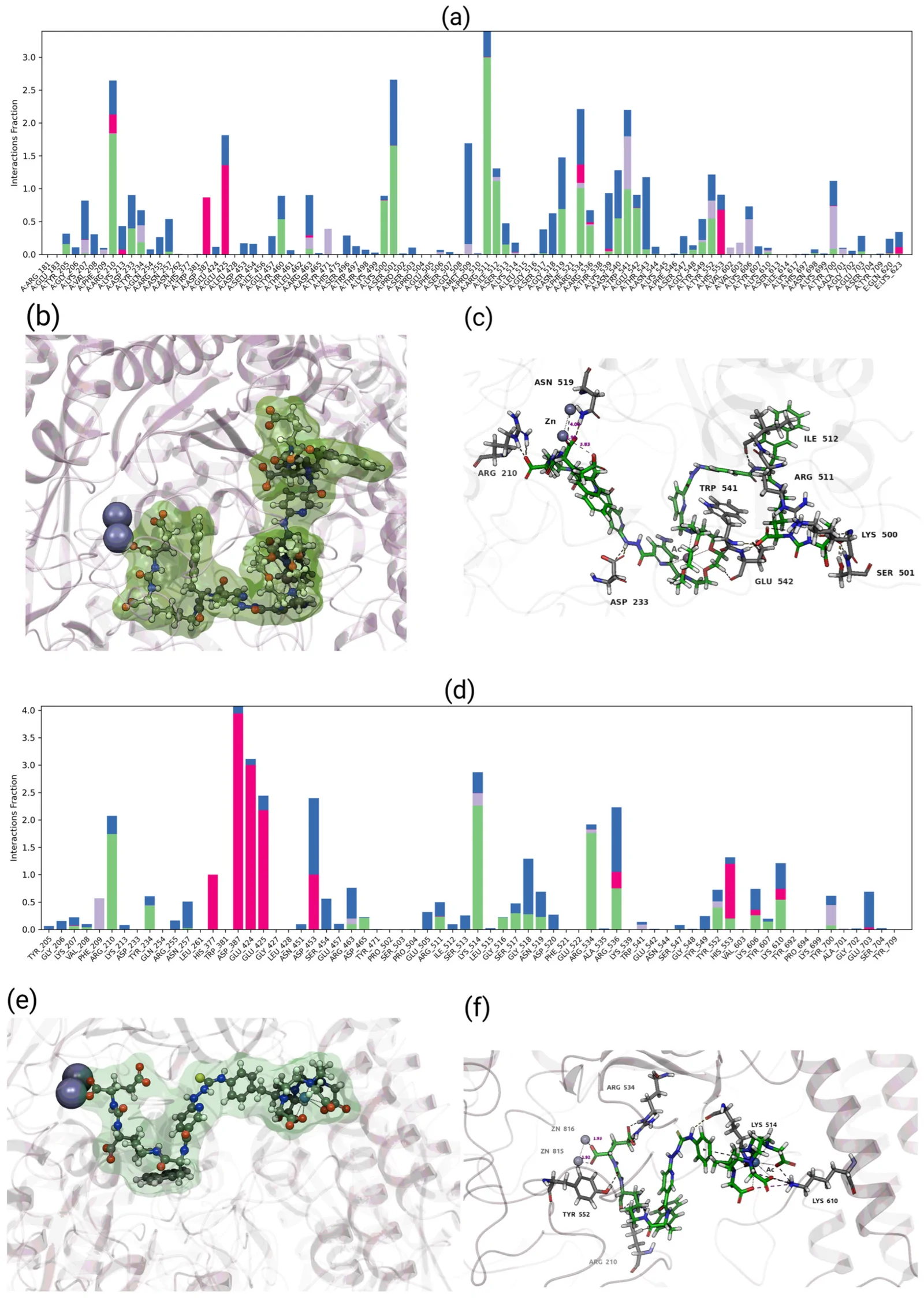
Molecular Dynamics Simulation System. (**a**) Fractional occupancy of protein–ligand contacts between PSMA and M-iPSMA over 500 ns. (**b**) [^225^Ac]Ac-M-iPSMA at the PSMA binding site. Side view. (**c**) Three-dimensional ligand interaction of [^225^Ac]Ac-M-iPSMA at the iPSMA binding site. (**d**) Fractional occupancy between PSMA and D-iPSMA over 500 ns. (**e**) [^225^Ac]Ac-D-iPSMA at the PSMA binding site. Side view. (**f**) Three-dimensional ligand interaction of [^225^Ac]Ac-D-iPSMA at the PSMA site. Distances between Zn^2+^ at the catalytic core and ligand’s key carbonyl oxygens are shown. Protein as ribbon; Zn^2+^ (purple), ligand (green), and Ac^3+^ (dark gray in chelators) as ball-and-stick. Protein-ligand contacts; H-bonds are displayed in green, hydrophobic in gray, ionic in pink and water bridges in blue.

RMSF analysis of D-iPSMA–PSMA over 500 ns shows most residues fluctuating ~0.8–2.5 Å; higher fluctuations (~4 Å) beyond residue ~600 are confined to solvent-exposed loops, while active-site residues show low mobility. Protein–ligand contact analysis confirms multi-anchor binding (Figure 3) with conserved residues ARG210, GLU424/425, ARG534, TYR552, and TYR700. D-iPSMA forms concentrated electrostatic interactions, while M-iPSMA shows a dispersed contact footprint due to its larger structure.

Metadynamics estimated the binding free energy (ΔG_bind_) (Table 2) and examined unbinding pathways. The free energy surfaces (FESs) (Figure S5) showed a global energy minimum at the bound state, with shallow escape barriers, indicating favorable binding kinetics. Two collective variables characterized unbinding: (CV1) the distance from the Zn^2+^ ion to the ligand’s carbonyl oxygen, reflecting disruption of metal coordination, and (CV2) the distance between the ligand’s center of mass (COM) and the binding pocket, indicating ligand displacement. For M-iPSMA and D-iPSMA, high-mass radiometal cores significantly influence the COM descriptor, providing a geometric perspective distinct from that of HYNIC-iPSMA and inhibitor 9OT (PSMA-1007).

HYNIC-iPSMA showed the most favorable ΔG_bind_, while M-iPSMA and D-iPSMA reflect the structural and topological complexity of their chelator–radiometal frameworks. For M-iPSMA, dual stabilization preserves Zn^2+^–carbonyl coordination (CV1) while the Ac^3+^–MACROPA core extends toward the vestibule, modulating net ΔG_bind_. D-iPSMA shows similar FES modulation via *p*-NCS-Bn-DOTA. The PSMA active site tolerates high-molecular-weight constructs while preserving Zn^2+^-mediated anchoring, supporting actinium-labeled conjugates for TAT.

### 2.3. Synthesis and Chemical Characterization

Fourier Transform Infrared Spectroscopy (FT-IR) confirmed successful conjugation in both systems. In M-iPSMA, amide bond formation was evidenced by the appearance of the characteristic Amide I (~1660 cm^−1^, ν C=O) and Amide II (~1540 cm^−1^, δ N–H + ν C–N) bands, absent in free MACROPA, together with the shift in the carboxylic acid ν C=O band (~1720 cm^−1^) toward lower wavenumbers. The transformation of the two primary NH_2_ stretching bands into a single secondary N–H band corroborated consumption of the free amino group upon conjugation (see pages S6, S7, Figures S8 and S9).

In D-iPSMA, thiourea bond formation was confirmed by the complete disappearance of the isothiocyanate band (~2050–2100 cm^−1^), accompanied by the appearance of the ν C=S stretching band (~1250–1350 cm^−1^), diagnostic of the thiourea linkage (see pages S6, S7, Figures S11 and S12).

The proton nuclear magnetic resonance (^1^H NMR) characterization (500 MHz, DMSO-d6) of HYNIC-iPSMA (see pages S6, S7, Figure S6), MACROPA-iPSMA, and DOTA-iPSMA relied on stacked-spectrum comparison and relative integration anchored to diagnostic pharmacophore signals: the urea NH protons (δ ≈ 6.3 ppm) and the most deshielded α-CH (δ ≈ 4.7 ppm). The connector pyridine was assigned unambiguously through cross-validated coupling constants. For MACROPA-iPSMA (Figure S7), the urea NH multiplet integrated for 4H (vs. 2H in HYNIC-iPSMA), confirming two intact iPSMA units on the macrocycle. The four diacylhydrazide NH groups appeared as singlets (δ 10.8, 10.3, 9.2, 9.1 ppm). For DOTA-iPSMA (Figure S10), the intact monomeric pharmacophore (urea NH = 2H) was confirmed and the DOTA macrocycle identified from N-CH_2_-COOH/cyclen signals at δ 3.0–3.6 ppm.

### 2.4. Radiochemical Purity and Stability

The initial radiochemical purity (RCP) of [^225^Ac]Ac-M-iPSMA was 98.5 ± 1.5% (n = 3), and that of [^225^Ac]Ac-D-iPSMA was 96.7 ± 0.3% (n = 3). After purification via Sep-Pak C18 solid-phase extraction, RCP was considered 100% as established by radio-HPLC with C18 reversed-phase elution. Retention times were 3.0–3.5 min for free ^225^AcCl_3_, and 14.0–14.5 min for both radiolabeled products.

Radiochemical stability studies demonstrated important differences between the two compounds. [^225^Ac]Ac-M-iPSMA retained 96.5% RCP at 24 h and 92.3% at 5 days, confirming the superior complex stability of the MACROPA chelator. In contrast, ^225^Ac-D-iPSMA showed an RCP of 67.2% at 24 h and 61.9% at 48 h, consistent with the slower complexation kinetics and lower thermodynamic stability of DOTA under physiological conditions. After 10 days, a new peak at 6–7 min appeared in [^225^Ac]Ac-M-iPSMA samples (attributed to [^225^Ac]Ac-MACROPA), reducing RCP to 74.8%.

To assess complex stability adequately, formulation parameters such as radiolytic scavengers and pH control must be carefully addressed. Including ascorbic acid/sodium ascorbate in the formulation medium does not compromise the stability of ^225^Ac-DOTA complexes; instead, it acts as a dual-purpose agent, serving as both a mild buffering system and a powerful radiolytic stabilizer [31]. Under these controlled conditions (typically at adjusted pH ranges of 5.5–6.5), the macrocyclic ^225^Ac-DOTA structure remains highly stable and inert, avoiding acid-induced dissociation while effectively quenching reactive oxygen species (ROS) generated by high-energy alpha particles [29]. The recent literature confirms that adding ascorbate quenchers maintains radiochemical purity (RCP) >90% for up to 24–48 h in ^225^Ac-DOTA peptide formulations [29,30], showing that an ascorbic acid-containing matrix optimally protects radioligand integrity without accelerating complex degradation.

Human serum protein binding of [^225^Ac]Ac-M-iPSMA was 1.9%, 4.5%, and 7.46% at 0.5, 2.0, and 24 h, respectively, while [^225^Ac]Ac-D-iPSMA showed 6.5%, 8.63%, and 14.9% at the same time points. The low serum protein binding of [^225^Ac]Ac-M-iPSMA is consistent with its more hydrophilic MACROPA chelator and is expected to minimize albumin-related pharmacokinetic modifications. Protein binding, particularly to human serum albumin, can modify the toxicity of radiopharmaceuticals by influencing their uptake and internalization at the intended target site in a living organism [32]. In cases where tumor accumulation is limited by slow uptake kinetics, the incorporation of albumin-binding moieties into PSMA-targeting radioligands has been shown to prolong blood circulation time, enhance tumor retention, and improve tumor-to-background ratios, ultimately increasing therapeutic efficacy [33]. In this study, the slow serum protein binding of both radiopharmaceuticals provided a substantial free fraction available for receptor-mediated tumor targeting, facilitating biodistribution to PSMA-expressing tissues and contributing to the favorable tumor uptake observed.

### 2.5. PSMA Expression

**Figure 4:**
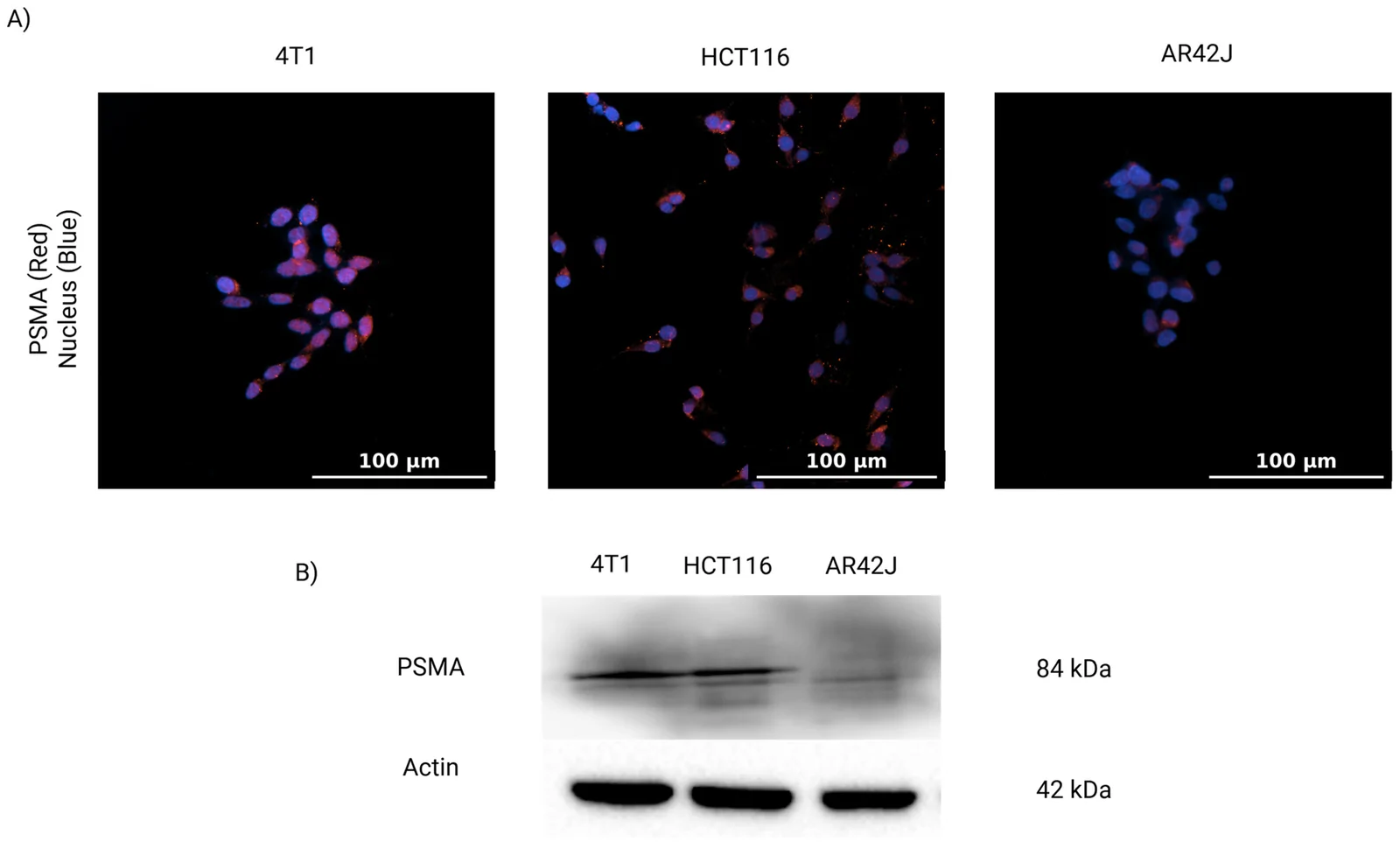
Differential expression of prostate-specific membrane antigen (PSMA) in cancer cells. (**A**) Immunofluorescence microscopic image of breast cancer (4T1), colorectal cancer (HCT116), and pancreatic cancer (AR42J) cells. PSMA expression is shown in red and nuclei in blue. The PSMA-associated red signal was mainly localized at the plasma membrane/cell surface, with possible perimembranous or cytoplasmic signal compatible with receptor internalization and intracellular trafficking after fixation and permeabilization. (**B**) PSMA expression identified by Western blot. Actin expression is shown as a loading control.

The 4T1 cell line is derived from the BALB/cfC3H mouse strain. It represents a model of triple-negative breast cancer, lacking expression of the estrogen receptor, progesterone receptor, and human epidermal growth factor receptor 2 (HER2). Shirke et al. (2024) [34] demonstrated PSMA and CD31 expression in both primary and metastatic tumors established from 4T1 cells in a syngeneic mouse model. Consistent with these findings, Figure 4 shows differential PSMA expression in cells from different cancer types. In both 4T1 triple-negative breast cancer cells and colorectal cancer cells, the PSMA protein is stained red. In contrast, a lower protein concentration is observed in AR42J pancreatic cancer cells. These results can be corroborated by Western blot analysis, which shows a much lower PSMA protein concentration in the AR42J cell line compared to 4T1 and HCT116. The PSMA messenger RNA transcript encodes a 750-amino acid protein with a predicted molecular weight of approximately 84 kDa. Nevertheless, extensive post-translational N-linked glycosylation increases the apparent molecular weight of the mature protein to over 100 kDa [35]. Accordingly, PSMA was detected by Western blot at an approximate molecular weight of 84 kDa (unglycosylated form), consistent with its predicted core polypeptide. Glycosylation is required for the enzymatic activity of PSMA, which is expressed on the apical cell surface in both monomeric and dimeric forms [35]. The PSMA expression was 4T1 > HCT116 > AR42J.

### 2.6. Uptake and Internalization

Using purified radiopharmaceuticals for in vitro evaluation, two-way ANOVA (α = 0.05) revealed a significant effect of cell line (Column Factor: *p* < 0.0001) on ^225^Ac-iPSMA uptake, while chelator identity (Row Factor: *p* = 0.9879) and the interaction between factors (*p* = 0.6795) were not significant. Tukey’s post hoc comparisons against ARJ42 showed that uptake of ^225^Ac-M-iPSMA was significantly higher in HCT116 (5.20 ± 0.45%/5 × 10^5^ cells; *p* = 0.0146) and 4T1 (9.41 ± 1.98%/5 × 10^5^ cells; *p* < 0.0001) compared with ARJ42 (1.23 ± 0.83%/5 × 10^5^ cells). Similarly, ^225^Ac-D-iPSMA uptake was significantly higher in 4T1 (9.13 ± 2.59%; *p* = 0.0002) compared with ARJ42 (2.09 ± 0.32%), while the increase in HCT116 (4.65 ± 0.97%) relative to ARJ42 did not reach significance (*p* = 0.1185).

For internalization, two-way ANOVA likewise showed a significant effect of cell line (*p* < 0.0001) with no effect of chelator (*p* = 0.6815) or interaction (*p* = 0.1009). Tukey’s post hoc comparisons against ARJ42 showed that both conjugates had significantly lower internalization in ARJ42 (0.83 ± 0.46 and 0.77 ± 0.56%/5 × 10^5^ cells for M-iPSMA and D-iPSMA, respectively) than in HCT116 (2.93 ± 0.32%, *p* = 0.0021; 3.57 ± 0.66%, *p* = 0.0002) and 4T1 (4.88 ± 0.87%, *p* < 0.0001; 3.96 ± 0.40%, *p* < 0.0001).

**Figure 5:**
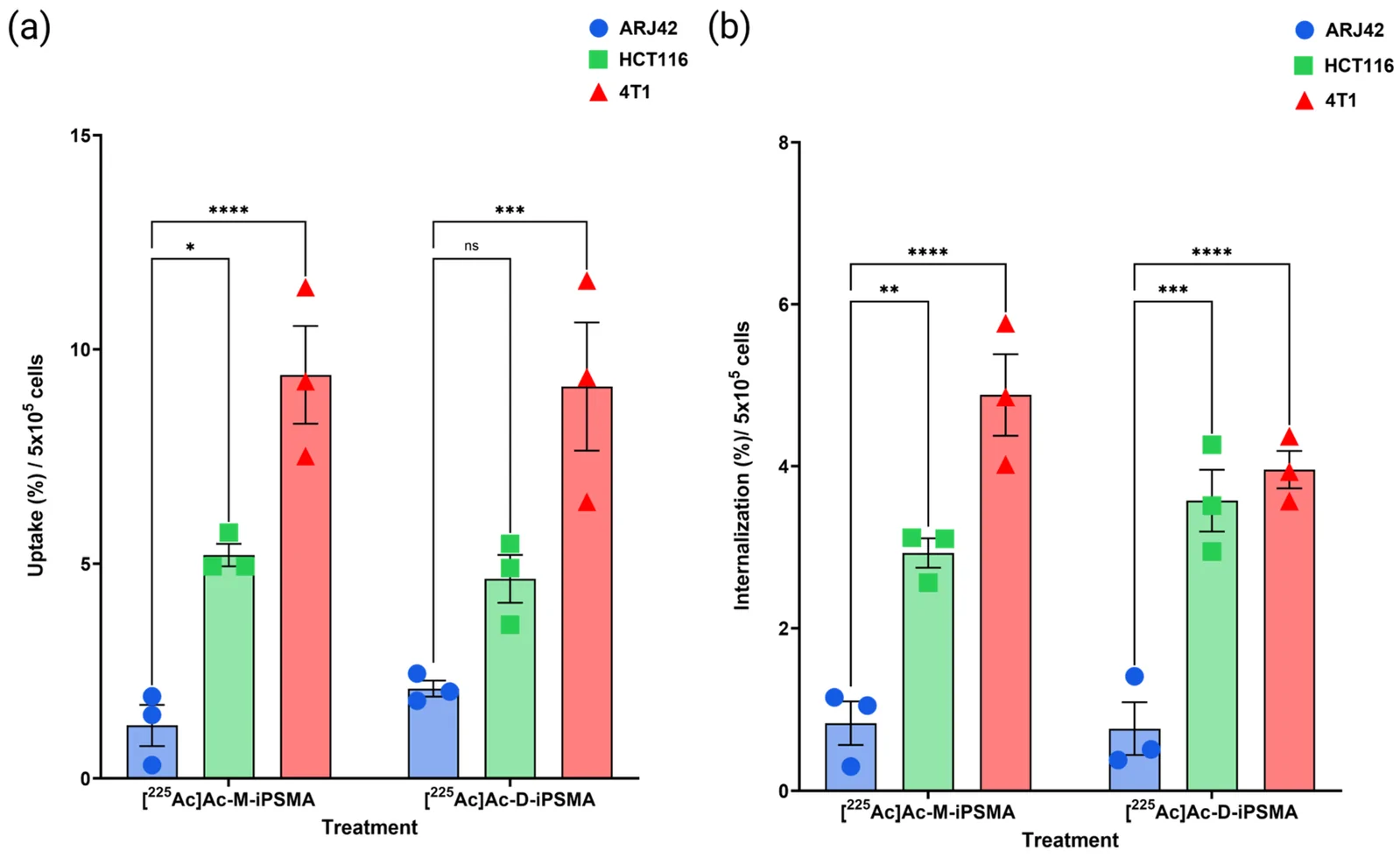
Uptake and internalization of [^225^Ac]Ac-M-iPSMA and [^225^Ac]Ac-D-iPSMA in 4T1 (PSMA-positive), HCT116 (PSMA-positive) and AR42J (PSMA-negative) cells. (**a**) Cell uptake (%/5 × 10^5^ cells). (**b**) Internalization (%/5 × 10^5^ cells). Statistical differences were analyzed by two-way ANOVA followed by Tukey’s multiple comparisons test (α = 0.05). * *p* < 0.05, ** *p* < 0.01, *** *p* < 0.001, **** *p* < 0.0001; ns, not significant.

These data demonstrate significantly higher PSMA-mediated uptake and internalization of [^225^Ac]Ac-M-iPSMA and [^225^Ac]Ac-D-iPSMA in PSMA-positive 4T1 and HCT116 cells compared to PSMA-negative AR42J baseline. Uptake observed in AR42J cells should be interpreted as nonspecific uptake and does not indicate PSMA-mediated internalization (Figure 5). Blocking studies further confirmed PSMA-mediated uptake: pre-incubation of 4T1 cells with excess HYNIC-iPSMA reduced uptake of [^225^Ac]Ac-M-iPSMA by 25.14%.

### 2.7. Cell Saturation Binding Assay

**Figure 6:**
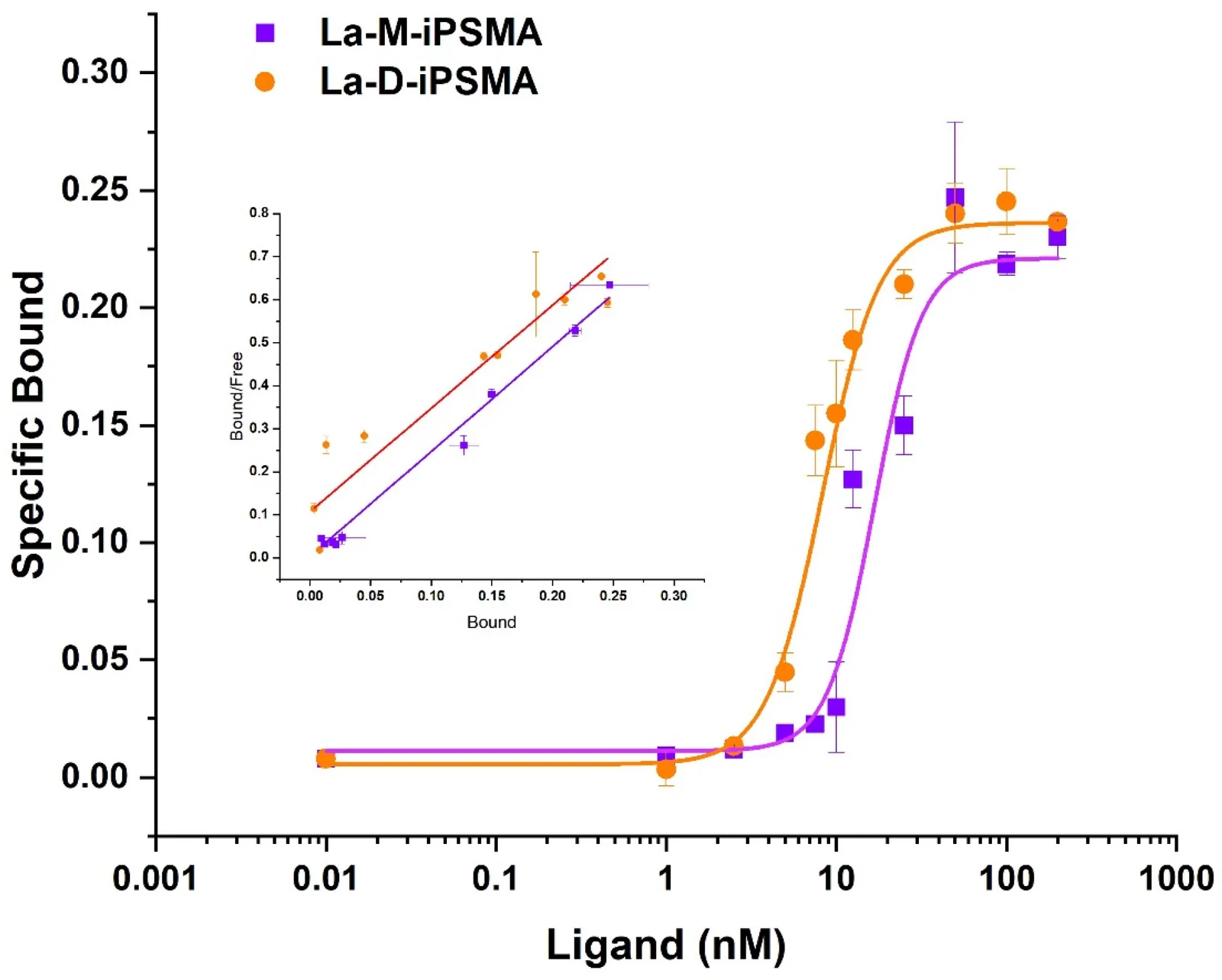
Binding affinity plot with main graph (semi-logarithmic) and Scatchard inset of La-M-iPSMA ana-D-iPSMA.

Saturation binding analysis showed nanomolar-range binding affinity (Figure 6). La-M-iPSMA yielded Kd = 16.76 ± 2.51 nM (R^2^ = 0.9815) and B_max_ = 0.221 by nonlinear regression using the Hill equation. La-D-iPSMA yielded Kd = 8.28 ± 0.51 nM (R^2^ = 0.998) and B_max_ = 0.2362. Both Hill coefficients were greater than 1, indicating positive cooperativity.

Scatchard analysis produced a positively sloped, linear plot (linear slope = 2.39 ± 0.85 and 2.43 ± 0.11) for La-M-iPSMA and La-D-iPSMA, respectively, diagnostic of positive homotropic cooperativity. The upward curvature of the Scatchard plot is consistent with a receptor system in which ligand binding at one site increases affinity at remaining sites. The saturation binding curve, Hill coefficient, positive Scatchard slope, and Hill plot slope all converge on the same interpretation: the binding interaction exhibits positive cooperativity, with apparent K_d_ 16.76 nM and 8.28 nM. Nanomolar-range binding to PSMA has been reported for several radioligands: 23.38 nM and 11.56 nM for [^225^Ac]Ac-mcp-M-alb-PSMA (^225^Ac-Macropa-Monomer-albumin-PSMA) and [^225^Ac]Ac-mcp-D-alb-PSMA (^225^Ac-Macropa-Dimer-albumin-PSMA), respectively, or [^225^Ac]Ac-mcp-M-PSMA and [^225^Ac]Ac-mcp-D-PSM (30.83 nM and 10.20 nM) using PSMA-positive LNCaP cells [21]. These comparisons support the clinical and preclinical relevance of the nanomolar K_d_ values obtained here, because PSMA radioligands with affinities in this range have been used for receptor-mediated imaging and therapy applications [21,23,24,25,26,27,28,31]. The K_d_ = 8.28 ± 0.51 nM (La-D-iPSMA) and Kd = 16.76 ± 2.51 nM determined here thus fall within the range of clinically and preclinically explored PSMA-targeting agents, indicating high enough intrinsic affinity for specific receptor engagement at therapeutic concentrations.

### 2.8. Apoptosis and DNA Damage

**Figure 7:**
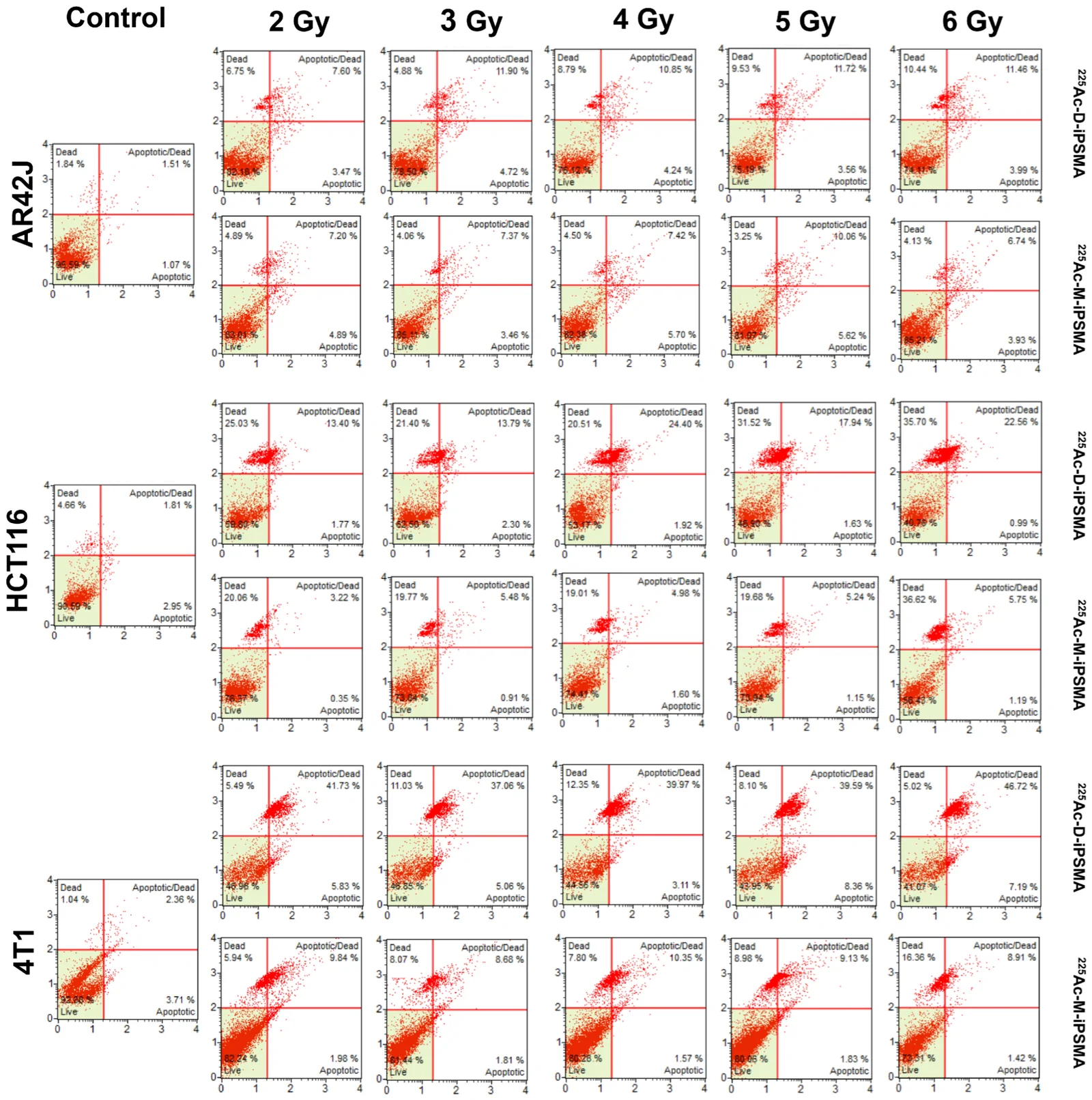
Apoptosis assessment of AR42J, HCT116 and 4T1 cell lines treated with doses of 2 Gy to 6 Gy of [^225^Ac]Ac-D-iPSMA and [^225^Ac]Ac-M-iPSMA. Treatment with [^225^Ac]Ac-D-iPSMA significantly increased the percentage of 4T1 cells in late apoptosis (apoptotic/dead) Caspase 3/7 (+) 7-AAD (+). Cells without any treatment serve as a negative control.

To analyze the percentage of apoptosis and cell death of cancer cell lines after radiopharmaceutical exposure, the Muse Caspase 3/7 kit (Catalog No. MCH100108) was used. Apoptosis was assessed through activated Caspase 3/7. Caspases (cysteinyl-directed aspartate-specific proteases) are cysteine proteases that play a central role in the activation of programmed cell death. Relative percentages of apoptotic cells exhibiting Caspase-3/7 activity (+) and 7-AAD (−) and Late Apoptotic/Dead cells exhibiting Caspase −3/7 (+) and 7-AAD (+) are shown in Figure 7.

At 2 Gy, [^225^Ac]Ac-D-iPSMA induces 4.3-fold greater late apoptosis than [^225^Ac]Ac-M-iPSMA (41.74% vs. 9.8%) in triple-negative breast cancer cells (4T1). This difference is consistent with the superior PSMA binding affinity of D-iPSMA (K_d_ = 8.28 ± 0.51 vs. 16.76 ± 2.51 nM for M-iPSMA), which results in a higher absorbed dose at equivalent administered activity. In contrast, only 7.2% of the pancreatic cell line AR42J undergoes apoptosis (Figure 7). The [^225^Ac] Ac-M-iPSMA also increases the percentage of cells in apoptotic death in 4T1 and HCT116 (colorectal cancer cell line), 8.9% and 5.7%, respectively, at 6 Gy (Figure 7). Similarly, the epidermal growth factor receptor-targeted antibody macropa-tzPEG3Sq-ch806, radiolabeled with ^225^Ac, significantly reduces cell proliferation and promotes apoptosis in preclinical models of glioblastoma and colorectal cancer following a single dose of the radiopharmaceutical (18.5 kBq, 0.5 µg) [36].

**Figure 8:**
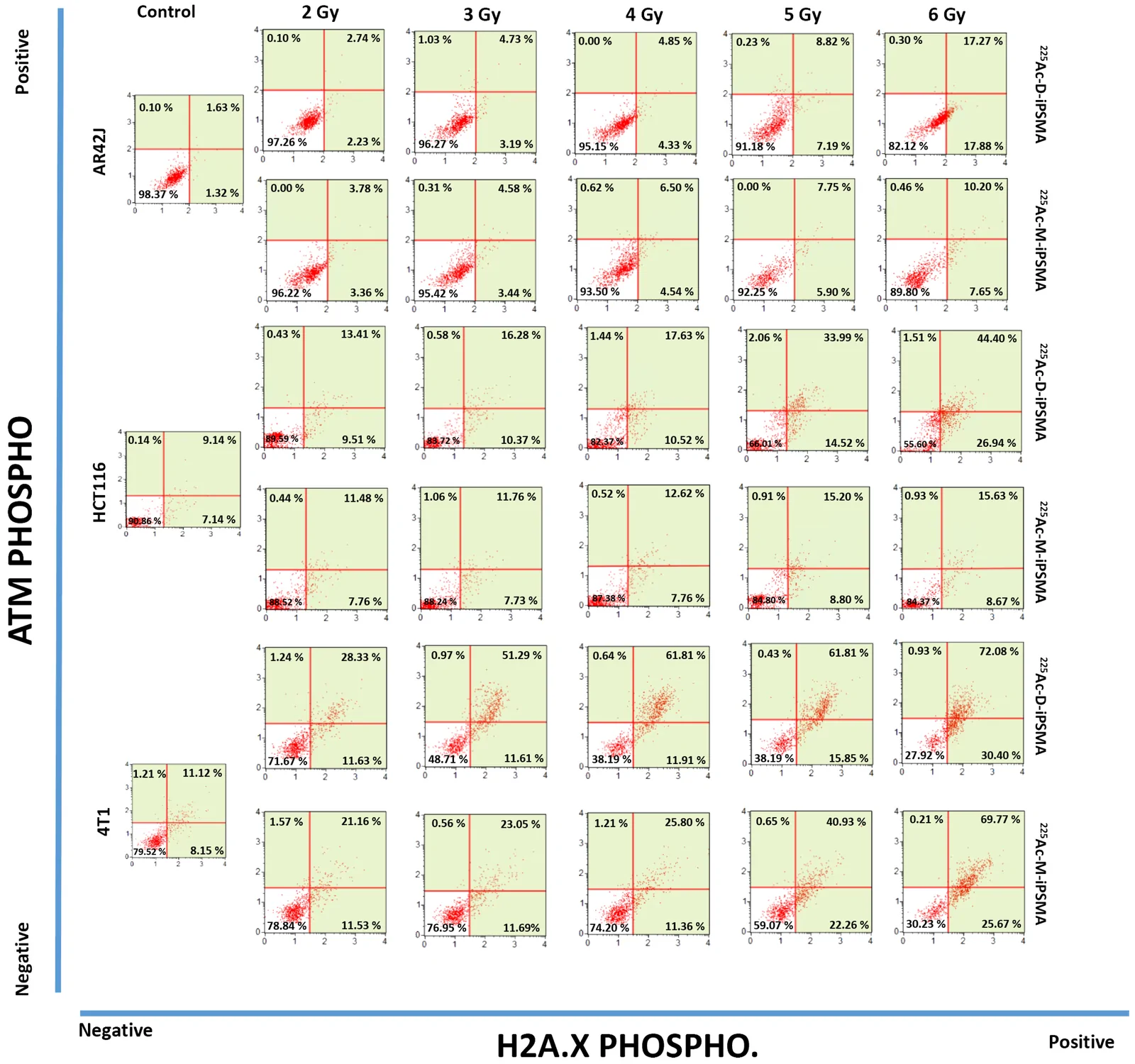
DNA damage analysis. AR42J, HCT116 and 4T1 cell lines were treated with different doses of [^225^Ac]Ac-D-iPSMA and [^225^Ac]Ac-M-iPMSA. Dual labeling with phospho-ATM and γ-H2AX serves as a biomarker of DNA damage. Triple-negative breast cancer 4T1 cells exhibited a higher percentage of DNA-damaged cells after treatment with both radiopharmaceuticals at a dose of 6 Gy.

Ionizing radiation induces cell death in tumor cells through DNA damage. Flow cytometric quantification of phosphorylated histone γ-H2A.X correlates with the number of DNA strand breaks, cell death, and radiosensitivity. In this study, a Muse Multi-Color DNA Damage Kit was used to quantify the percentage of cells positive for dual phospho-ATM and γ-H2AX labeling, which strongly indicates DNA damage. The [^225^Ac]Ac-D-iPSMA induced a significantly higher percentage of DNA-damaged cells in the 4T1 cell line (72.08%) at 6 Gy, correlating with the percentage of apoptotic cells. Similarly, [^225^Ac]Ac-M-PSMA promoted a 69.77% increase in DNA-damaged cells (Figure 8). In contrast, at a 6 Gy dose of [^225^Ac]Ac-D-iPSMA, AR42J and HCT116 cells showed increases of only 17.27% and 44.40%, respectively, while treatment with [^225^Ac]Ac-M-iPSMA resulted in increases of 10.20% and 15.63% in AR42J and HCT116 cells, respectively (Figure 8). Likewise, the radiopharmaceutical ^157^Gd-DOTA-PSMA used for Gadolinium-based electron capture therapy significantly increases γ-H2AX expression in PSMA-overexpressing prostate cancer cells [37].This comparison is included only to highlight γ-H2AX as a biomarker of radiation-induced DNA damage and does not imply mechanistic similarity between Ga neutron capture therapy and TAT.

Breast cancer cells present a complex scenario where high baseline apoptosis suggests a response, but limited radiation enhancement indicates potential resistance mechanisms. The differential radiosensitivity suggests that HCT116 cells would be most responsive to radiation therapy, while AR42J and 4T1 cells showed greater resistance to alpha radiation, potentially requiring higher doses or combination treatments.

The high proportion of early apoptotic cells in all evaluated radiation doses suggests that 4T1 cells maintain active apoptotic pathways even under stress.

### 2.9. Phosphokinase Profiling

**Figure 9:**
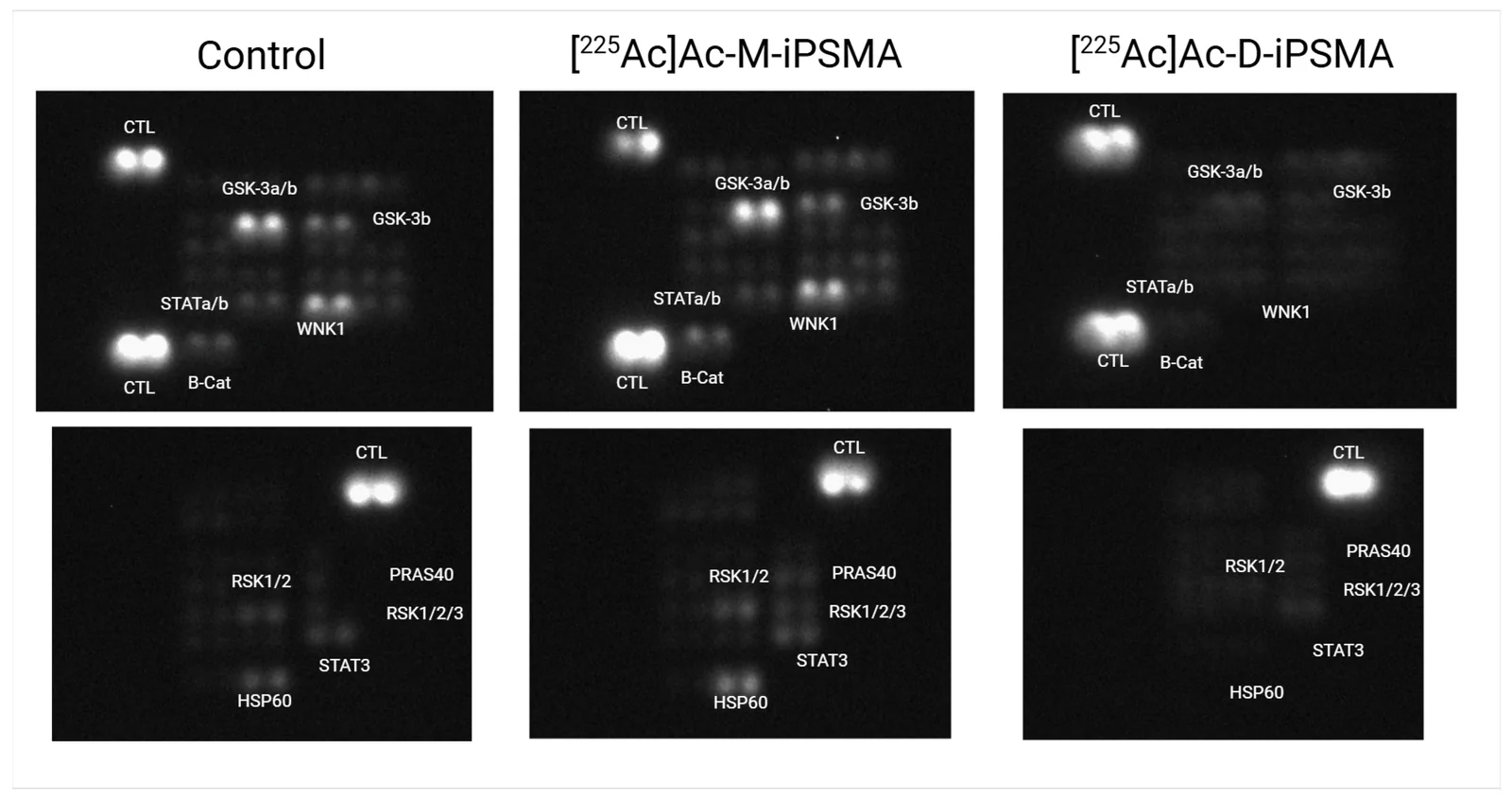
Effect of alpha-based radiopharmaceuticals [^225^Ac]Ac-M-iPSMA and [^225^Ac]Ac-D-iPSMA in human phosphokinase of breast cancer cells. Left panel, phosphokinase array of 4T1 cancer cells without treatment (control). Middle panel, phosphokinase array of 4T1 cancer cells after 4Gy of [^225^Ac]Ac-M-iPSMA. Right panel, phosphokinase array of 4T1 cancer cells after 4Gy of [^225^Ac]Ac-D-iPSMA.

The efficacy of PSMA-targeted radioligands has been widely reported and experimentally confirmed; however, there is limited information about their cellular and molecular effects. For instance, [^177^Lu]Lu-PSMA-617 has been reported to inhibit cell proliferation through regulatory mechanisms involving cyclin D1 and cyclin E1 and by increasing the cyclin-dependent kinase inhibitor p21, ultimately causing cell-cycle arrest [37]. Following exposure to [^225^Ac]Ac-D-iPSMA, reduced phosphorylation of GSK-3α/β at S21/S9 was observed (Figure 9). This kinase has been implicated in tumor progression by stabilizing components of the β-catenin complex [38]. A decrease in WNK1 phosphorylation was also observed after [^225^Ac]Ac-D-iPSMA treatment (Figure 9). WNK kinases contribute to tumor growth, metastasis, and angiogenesis through downstream kinase substrates such as SPAK and OSR1 [39]. The observed decrease in β-catenin levels in 4T1 cells may be related to the treatment-associated modulation of these pathways [40], but additional experiments are required to confirm causality. One of the proteins differentially expressed between treatments was HSP60. Following treatment with [^225^Ac]Ac-M-iPSMA, HSP60 levels increased compared with the control; in contrast, treatment with [^225^Ac]Ac-D-iPSMA resulted in reduced HSP60 expression (Figure 9). Loss or instability of HSP60 has been associated with p53-mediated pro-apoptotic responses [41]. Accordingly, treatment with [^225^Ac]Ac-M-iPSMA may promote therapeutic resistance, as evidenced by a lower percentage of cells exhibiting DNA damage than with [^225^Ac]Ac-D-iPSMA treatment. In contrast, [^225^Ac]Ac-D-iPSMA is associated with decreased HSP60 levels, increased DNA damage, and enhanced apoptotic cell death. The difference in kinase activation observed between the two radiopharmaceuticals correlates with the extent of apoptosis and DNA damage-induced cell death. It was then hypothesized that differences in the binding affinity of the chelating moieties may explain this effect. Structural modifications introduced between the PSMA-binding motif and the DOTA radiometal chelator have been shown to enhance binding affinity and tumor retention and to improve overall pharmacokinetic performance [42]. Moreover, PSMA has been implicated not only as a target for radioligand delivery but also as an active participant in intracellular signaling. The intracellular domain of PSMA interacts with the cytoskeletal protein filamin A as part of a macromolecular signaling complex that includes integrin β1, Crk-associated substrate (p130Cas), c-Src, and the epidermal growth factor receptor (EGFR). Assembly of this complex promotes activation of the protein kinase B (AKT) and mitogen-activated protein kinase (MAPK) signaling pathways, thereby enhancing cell proliferation, survival, and tumor progression. Therefore, in addition to DNA damage induced by α-particle irradiation, the molecular effects observed after ^225^Ac-D-iPSMA treatment may also be associated with disruption of PSMA-mediated signaling, which could contribute to inhibition of pro-survival pathways and induction of apoptosis [35]. The potential contribution of chelator-related binding differences and PSMA-mediated signaling should be considered hypothesis-generating and requires further validation.

### 2.10. Clonogenic Assay

**Figure 10:**
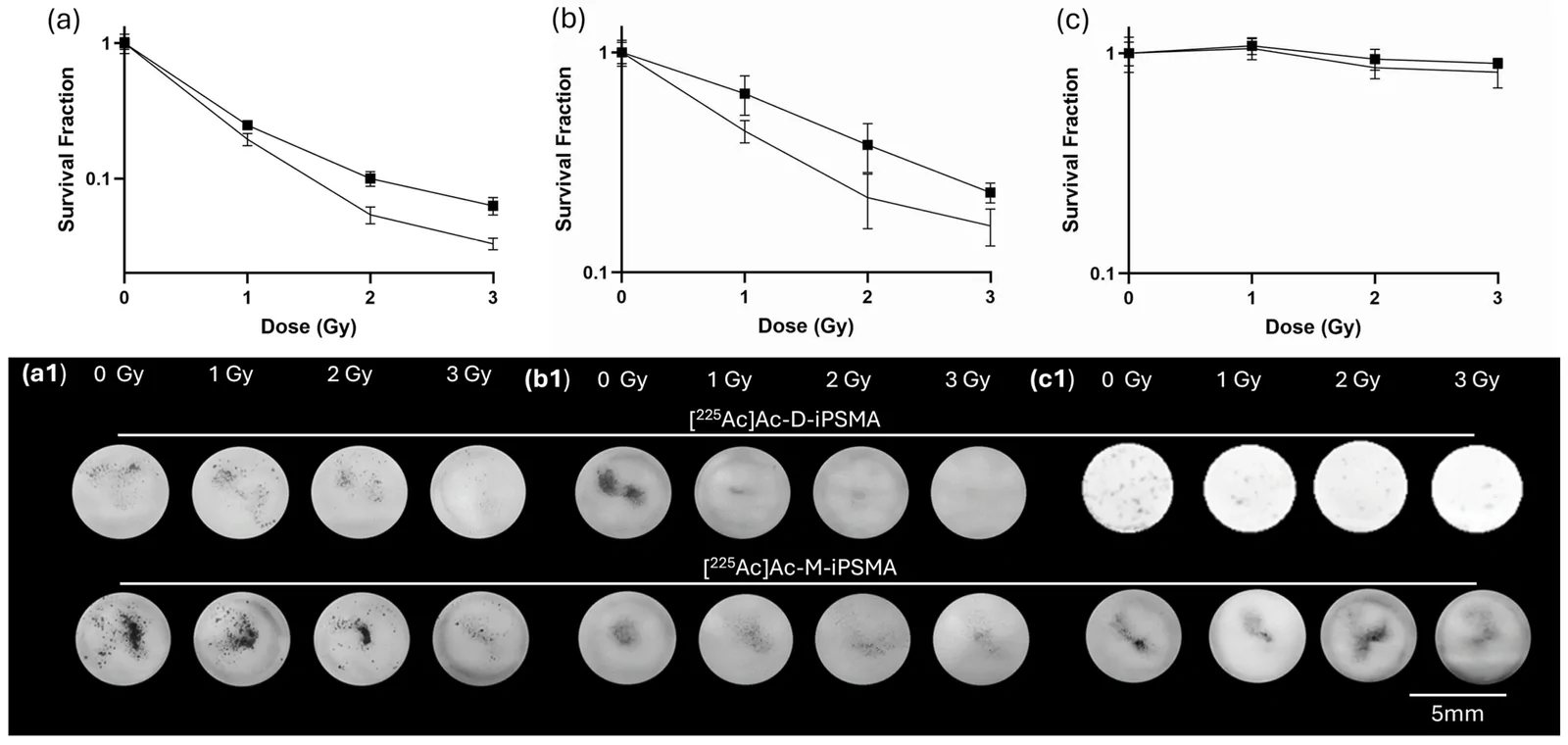
Survival curves of (**a**) 4T1, (**b**) HCT-116, and (**c**) AR42J cells treated with increasing radiation doses of ([225Ac]Ac-M-iPSMA) dimer (line + symbol) compared to [225Ac]Ac-D-iPSMA monomer (line). Representative images of colonies formed and counted to estimate surviving fraction. The lower (**a1**–**c1**) micrographs correspond to representative colony-formation fields for each cell line and treatment condition shown in the upper survival-curve panels. Median and standard deviation calculated from three measurements.

Clonogenic assays on the three cell lines (4T1, HCT116, and AR42J) showed a progressive, dose-dependent reduction in the survival fraction after exposure to increasing concentrations of the ([^225^Ac]Ac-M-iPSMA) dimer or the [^225^Ac]Ac-D-iPSMA monomer. In all three cell lines, the dose–survival relationship fit well to a linear–quadratic model, with the linear component dominant, as expected for alpha-emitters with high linear energy transfer (LET). Unlike low-LET radiation (photons, electrons), where the quadratic component is often relevant even at low doses, alpha particles predominantly induce double-strand breaks (DSBs) directly and independently of cellular repair [43], which explains the markedly linear decline in survival observed from low to intermediate doses. The alpha radiobiological component of the survival curves showed a lethality order consistent with the hierarchy of specific PSMA uptake across the three cell lines. 4T1 cells exhibited the highest α coefficient, associated with high PSMA density, which favors specific uptake of the monomeric radiopharmaceutical and results in a greater biological effect on cell survival per unit of administered activity. In the HCT116 cell line, the radiobiological response was intermediate, whereas in the AR42J cell line, which exhibited the lowest radiopharmaceutical uptake, the survival curve remained unchanged (flat), with no effect on cell survival even at the higher evaluated dose. A direct correlation was observed between the percentage of cell uptake and the survival fraction, consistent with the fundamental radiobiological principle of radionuclide-targeted therapy [44]. Survival and viable clone formation depend on the effective absorbed dose at the cellular level, which is determined by PSMA density and the efficiency of radiopharmaceutical internalization rather than by the initial amount of radiopharmaceutical administered in the culture medium. In all three cell lines, the monomeric radiopharmaceutical produced a greater reduction in the survival fraction (Figure 10, line) compared to the dimeric form (Figure 10, line + symbol), suggesting that the advantage of the monomer results from its superior binding and retention capacity.

### 2.11. ROS Production

Since [^225^Ac]Ac-M-iPSMA and [^225^Ac]Ac-D-iPSMA showed no statistically significant difference (Figure S13), [^225^Ac]Ac-M-iPSMA was selected to evaluate ROS production for the [^177^Lu]Lu and [^225^Ac]Ac-iPSMA tandem. Tandem or augmented targeted radionuclide therapy approaches combining ^177^Lu- and ^225^Ac-labeled PSMA ligands have been clinically investigated to balance efficacy and toxicity in advanced prostate cancer [45,46]. Simultaneous doses of [^177^Lu]Lu and [^225^Ac]Ac-iPSMA were administered to evaluate the oxidative response of the alpha–beta combination.

**Figure 11:**
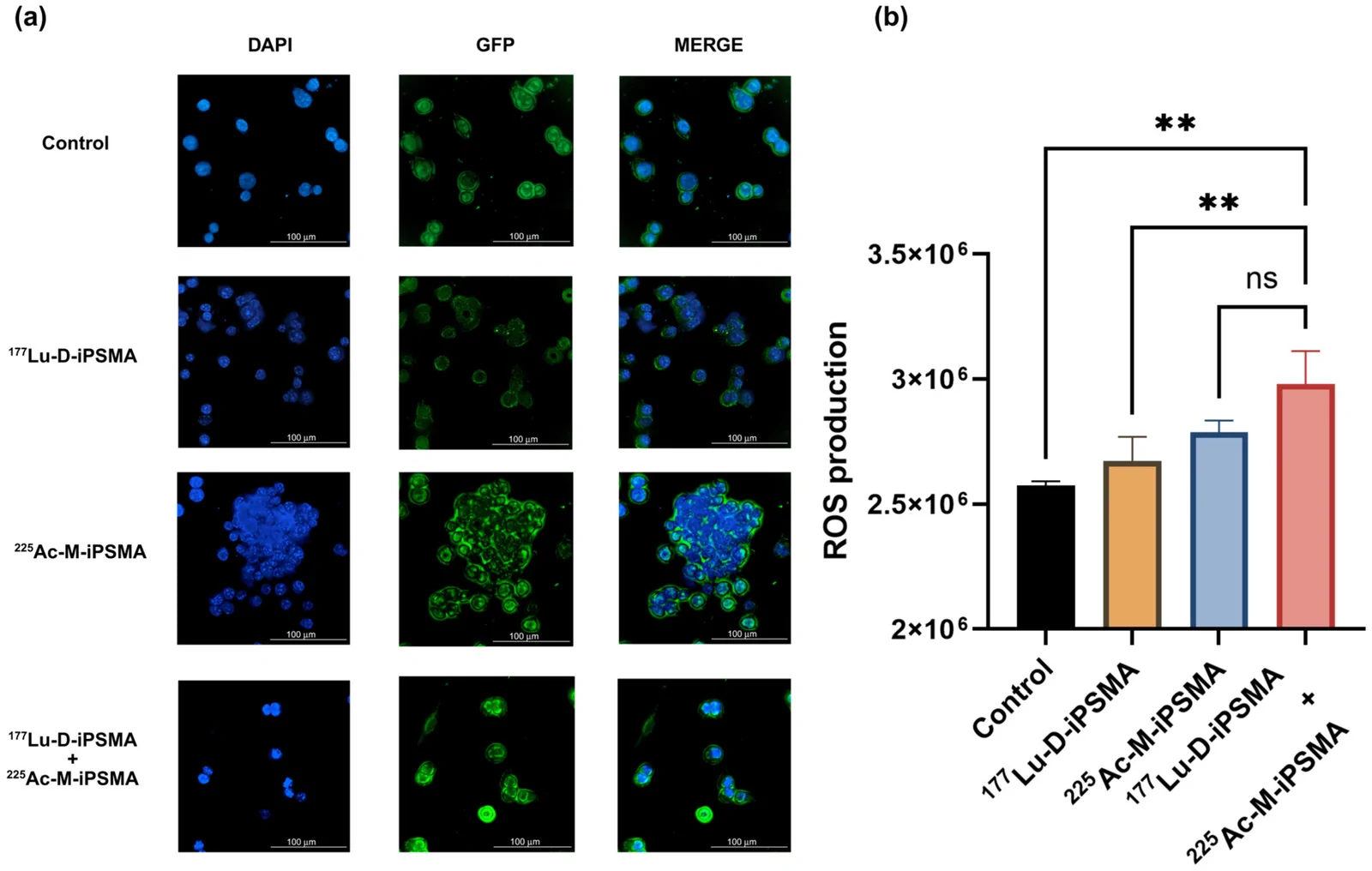
ROS generation patterns in 4T1 cells after treatment with 6 Gy of [^177^Lu]Lu-D-iPSMA, 6 Gy of [^225^Ac]Ac-M-iPSMA, and their combination (3 Gy each). (**a**) Representative fluorescence micrographs showing nuclear staining (DAPI, blue) and intracellular ROS accumulation detected (green). Scale bar = 100 µm. (**b**) Quantification of ROS production. Statistical differences were analyzed by ordinary one-way ANOVA followed by Dunnett’s multiple comparisons test (α = 0.05) using GraphPad Prism. ** *p* < 0.01; ns, not significant.

Figure 11 shows ROS generation patterns in 4T1 cells after treatment with 6 Gy of [^177^Lu]Lu-D-iPSMA, 6 Gy of [^225^Ac]Ac-M-iPSMA, and their combination (3 Gy each). Results showed increased ROS production across treatment groups, with the combination therapy achieving the highest levels (3 × 10^6^), about 15% above control and 9% over [^225^Ac]Ac-M-iPSMA monotherapy.

Figure 11 shows fluorescence micrographs of 4T1 cells after exposure to the evaluated treatments. The increased fluorescence signal is consistent with ROS accumulation under the evaluated conditions; however, cell clustering or increased fluorescence alone does not demonstrate radiation-induced bystander effects. Cell clustering was therefore described only as a morphological observation and was not used as evidence of a specific radiation-induced mechanism. [^225^Ac]Ac-M-iPSMA treatment showed intense GFP fluorescence, indicating ROS accumulation within cellular compartments. Any possible bystander effect should therefore be regarded only as a potential explanation that requires dedicated experimental confirmation. The short range of alpha particles (<100 µm) supports highly localized energy deposition within a few cell diameters [11,47].

The combination of [^177^Lu]Lu-iPSMA and [^225^Ac]Ac-M-iPSMA showed enhanced ROS production compared with monotherapy, suggesting potential synergistic mechanisms. This finding is consistent with emerging clinical evidence supporting tandem therapy approaches in targeted radionuclide therapy (TRT), which is defined here as the use of tumor-targeting molecules labeled with therapeutic radionuclides to deliver radiation selectively to disease-associated cells or tissues. Studies in metastatic castration-resistant prostate cancer have shown that combining [^177^Lu]Lu- and [^225^Ac]Ac-PSMA-617 can achieve biochemical partial remission in 53.3% of patients and molecular imaging partial remission in 66.7% of patients with poor prognosis [48,49].

Spatial and temporal effects may explain this synergy. Beta particles provide a crossfire effect that can irradiate the cells not directly targeted by the radiopharmaceutical, while alpha particles cause complex, irreparable DSBs oxygen-independently [45]. Furthermore, the enhanced ROS generation observed with combination therapy may reflect additive or synergistic oxidative stress that overcomes cellular antioxidant defenses. Cancer cells maintain ROS levels sufficient to promote proliferation and survival signaling, but not high enough to trigger cell death. The dual-radionuclide approach is more effective than either agent alone, enhancing therapeutic efficacy by engaging multiple cell death pathways, including apoptosis, necrosis, and ferroptosis. The demonstration that a PSMA-targeted radiopharmaceutical can induce substantial ROS generation in 4T1 cells suggests potential therapeutic utility, particularly given that PSMA expression has been documented in the tumor-associated neovasculature of various solid tumors, including breast cancer [46,50].

It is important to note that this study represents a single time point, but ROS levels are dynamic and subject to cellular regulation. Understanding the ROS production kinetics, peak levels, and duration of oxidative stress could inform optimal dosing and combination strategies [51].

### 2.12. Biokinetics and Dosimetry

The [^225^Ac]Ac-M-iPSMA biodistribution profile in 4T1-bearing mice indicated rapid blood clearance with hepatobiliary and renal elimination. The radiation absorbed dose in Table 3 showed the following order: tumor > spleen > liver > kidney > bone (Table 3).

Tumor-to-blood time-integrated activity (TIAC) indicates high tumor selectivity, and the tumor-to-kidney ratio (54:1) suggests a favorable therapeutic window. The radiopharmacokinetic model showed a rapid blood clearance followed by a terminal phase (57 min), favorable for minimizing hematological toxicity. This rapid clearance contrasts with albumin-binding PSMA conjugates, where [^225^Ac]Ac-mcp-D-alb-PSMA demonstrated prolonged blood retention of 8.44 ± 0.94% ID/g at 1 h post-injection and 2.84 ± 0.33% ID/g at 24 h [21]. The rapid blood clearance of standard [^225^Ac]Ac-PSMA-617 minimizes red marrow exposure, with reported doses of 0.05 Sv per MBq administered [13].

The liver model showed a rapid initial distribution (t_1/2_ ≈ 4 min), an intermediate phase (t_1/2_ ≈ 20.5 h), and a prolonged terminal phase (t_1/2_ = 20.6 h). The absorbed dose of 3.17 Gy/0.074 MBq (42.8 Gy/MBq) indicates moderate hepatic accumulation. This liver activity is interpreted as the combined result of hepatobiliary clearance of the radioconjugate, nonspecific hepatic uptake, and possible binding to PSMA expressed in hepatic or tumor-associated vasculature; it should not be interpreted as exclusively tumor-specific PSMA uptake. Additional blocking or metabolite studies would be required to determine the relative contribution of each mechanism.

The kidney model suggests initial rapid uptake followed by redistribution and slower clearance. The 1.62 Gy/0.074 MBq (21.9 Gy/MBq) indicates significant renal exposure. The renal uptake of PSMA-targeted agents reflects proximal tubular reabsorption of filtered radiopharmaceutical, a mechanism shared with other peptide-based radiopharmaceuticals. Recent image-based dosimetry studies of ^225^[Ac]Ac-PSMA I&T have reported kidney effective half-lives of 23–27 h indicating relatively rapid renal clearance that limits cumulative dose [52].

The low bone dose is favorable for minimizing hematological toxicity, as bone marrow is a critical dose-limiting organ for many radiopharmaceuticals. The elevated splenic absorbed dose can be attributed to PSMA expression in splenic vasculature and reticuloendothelial uptake.

The tumor model shows suitable retention with an intermediate phase (t_1/2_ = 6.9 h) and a slow phase (t_1/2_ = 239 h). The TIAC is about 12 times higher than that in blood and liver, indicating high PSMA expression and efficient internalization. The prolonged tumor retention is critical for maximizing therapeutic efficacy, as the 10-day physical half-life of ^225^Ac allows alpha-particle emission throughout the biological retention period. Effective half-lives for [^225^Ac]Ac-PSMA-I&T in lesions have been reported as 61 ± 55 h [52], supporting the extended retention captured in the terminal exponential phase of our model.

## 3. Materials and Methods

Radiochemicals: ^225^Ac(NO_3_)_3_ in solid form was obtained from Van Overeem Nuclear BV (Amsterdam, The Netherlands). ^177^LuCl_3_ n.c.a was obtained from ITM (Garching/Munich, Germany). MACROPA-NH_2_ was obtained from MCE (MedChemExpress, NJ, USA). Subsequent chemical synthesis of MACROPA-iPSMA was performed by Shanghai Yaxian Chemical Co., Ltd. (Jiading, Shanghai, China). p-SCN-Bn-DOTA (2,2′,2″,2‴,2⁗,2′′′′′-(2-(4-isothiocyanate benzyl)-1,4,7,10,13,16-hexaazacyclooctadecane-1,4,7,10,13,16-hexayl)hexaacetic acid) was obtained from Macrocyclics (Dallas, TX, USA). Dimethylformamide (DMF), diisopropylethylamine (DIPEA), and hydrochloric acid (99.999% trace metal basis) were purchased from Merck (KGaA, Darmstadt, Germany). The 2,3-bis-(2-methoxy-4-nitro-5-sulfophenyl)-2H-tetrazolium-5-carboxanilide) (XTT) kit was obtained from Roche Diagnostics (Indianapolis, IN, USA).

Cell lines 4T1, HCT116, and AR42J were purchased from ATCC^®^ (Manassas, VA, USA). Cell culture media RMPI-1640, Fetal Bovine serum, supplements, and antibiotics were purchased from Bovine & Biology (Toluca, Mexico).

### 3.1. In Silico Evaluation

#### 3.1.1. Protein Preparation and Molecular Dynamics

The PSMA structure was retrieved from the Protein Data Bank (PDB) entry 5O5T [53], which depicts the enzyme in complex with PSMA-1007 (9OT), a clinically approved inhibitor with a well-defined binding pose. In this crystal structure, 9OT binds to the PSMA active site, providing a high-quality reference for mapping the native binding pocket, setting grid coordinates, and confirming ligand–residue interaction profiles in both docking and molecular dynamics studies. Both structures were prepared using the Protein Preparation Workflow in Schrödinger Maestro 2024-4 v14.2.118 (Schrödinger LLC, NY, USA). Non-structural water molecules were removed, missing hydrogens were added, and protonation states were set at pH 7.4 with the PROPKA algorithm [54].

The PSMA structure was then subjected to molecular dynamics simulations (MDSs) using Maestro v2024-4 v14.2.118, MMshare Version 6.4.135, Release 2024-4, and Desmond Multisim v4.0.0 interoperability tools package under the OPLS-AA force field [55,56], to generate a representative conformational ensemble. Simulations were run under isothermal–isobaric (NPT) conditions at 310.15 K and 1 atm, with an octahedral water box of explicit TIP3P water molecules extending 12 Å from the solute and Na^+^/Cl^−^ counterions to neutralize the system charge. Selected frames were obtained via hierarchical clustering of alpha-carbon RMSD across trajectories. Ensemble docking was performed using GNINA (Graph Neural Network-based Docking Software) 1.1 [57]. The Vinardo scoring function was used with a pre-trained deep learning model (cnn_scoring = redock_default2018). PSMA1007 (9OT) served as the reference ligand, and the docking grid was defined at (center_x = −16.38, center_y = 4.88, center_z = −33.42) with the same box dimensions.

For the 5O5T system, the ligands MACROPA-iPSMA, DOTA-iPSMA, and HYNIC–iPSMA were evaluated against PSMA1007 (9OT) using exhaustiveness = 128, num_modes = 5, cpu = 24, replicas = 10, Monte Carlo search = enabled, and the same deep learning model. The highest-scoring poses from each docking run were selected for subsequent structural and statistical analyses.

#### 3.1.2. Molecular Dynamics Simulations

The best docked conjugates of each chelator were used as initial coordinates for MDSs to identify stable ligand–receptor binding modes. Simulations were performed using Maestro v2024-4 v14.2.118, MMshare Version 6.4.135, Release 2024-4, and Desmond Multisim v4.0.0 interoperability tools package (academic license) under the OPLS-AA force field [55,57]. The complex system was solvated in octahedral boxes of explicit TIP3P water molecules, extending 15 Å from the solute, and neutralized with Na^+^ or Cl^−^ ions. All MD simulations were carried out under periodic boundary conditions (PBCs). Initial energy minimizations were performed using a two-approximation scheme: 50,000 steps of steepest descent followed by 50,000 steps of conjugate gradient. Positional restraints (10 kcal·mol ^1^·Å^2^) were applied to the solute heavy atoms. The system was heated from 0 to 310 K over 100 ps under constant-volume conditions, followed by 5 ns of equilibration at 310 K with a relaxation time of 1 ps, using the Langevin thermostat. The Berendsen barostat was used to maintain a pressure of 1 atm with a relaxation time of 2 ps, and positional restraints were gradually reduced during equilibration. Production runs were performed for 500 ns under NPT conditions, using a 1 fs time step and constraints on hydrogen-bonded bonds. Long-range electrostatics were calculated with an 8 Å cutoff for non-bonded interactions.

Trajectory analysis was conducted using the Simulation Event Module of the Desmond–Maestro interoperability tools; alpha carbon root-mean-square deviation was calculated along complete trajectories to assess structural flexibility.

#### 3.1.3. Metadynamics Simulations

The free energy landscape associated with the binding and dissociation of selected inhibitors and PSMA (complexes) was explored using the well-tempered metadynamics (wt-MetaD) approach. This enhanced-sampling technique enables efficient exploration of rare events by accelerating transitions that are inaccessible on conventional molecular dynamics (MD) timescales [58].

All metadynamics simulations were carried out using Desmond v2024-4 (Schrödinger LLC), and trajectory post-processing and free-energy reconstruction were performed with the Metadynamics Analysis plugin implemented in Maestro. To characterize the ligand dissociation pathway, a collective variable CV1 was defined as the distance between the centers of mass (COMs) of the PSMA binding site and that of each ligand. The initial COM separation was approximately 3 Å from the Zn^2+^ metal center. This reaction coordinate was selected due to its direct physical relevance to the binding/unbinding of substrates [59].

Bias potentials in the form of Gaussian functions were deposited every 1 ps, using an initial height (ω) of 0.03 kcal·mol^−1^ and a Gaussian width (σ) of 0.5 Å. The bias factor parameters followed the standard wt-MetaD scheme, allowing progressive smoothing of the free-energy surface while preserving sufficient resolution in the vicinity of intermediate and transition states. Simulations were continued until either complete ligand dissociation was achieved or a maximum COM separation of 25 Å was attained. For each inhibitor, three independent metadynamics replicas of 50 ns were conducted to ensure statistical reliability and reproducibility. All simulations employed a 2 fs integration time step and were performed under isothermal–isobaric (NPT) conditions at 310 K and 1 bar, with the Nosé–Hoover thermostat and the Martyna–Tobias–Klein barostat, respectively. The potential of mean force (PMF) profiles were obtained by reconstructing the accumulated bias using time-dependent free-energy estimators. Convergence was assessed by monitoring stabilization of the deposited bias potential and consistency of PMF curves across independent replicas. Additional validation of the free-energy barriers was achieved by bootstrapping the final PMF across multiple trajectory segments, yielding robust estimates of the associated uncertainties.

#### 3.1.4. Structural and Statistical Analysis

Protein–ligand interaction analyses were visualized using the Ligand Interaction Diagram module in the Maestro suite, and three-dimensional representations were generated within the same environment. CNN-Docking scores (kcal·mol^−1^) were compiled for the three ligands in each system, and means ± standard deviations were calculated. Statistical analyses were performed using GraphPad Prism v10.4.1, with the nonparametric Kruskal–Wallis test used to compare relative binding affinities among complexes. A *p* < 0.05 threshold was considered statistically significant.

### 3.2. Synthesis of MACROPA-iPSMA (M-iPSMA) and p-NCS-Bn-DOTA-iPSMA (D-iPSMA)

#### 3.2.1. Synthesis of MACROPA-iPSMA (M-iPSMA)

**Figure 12:**
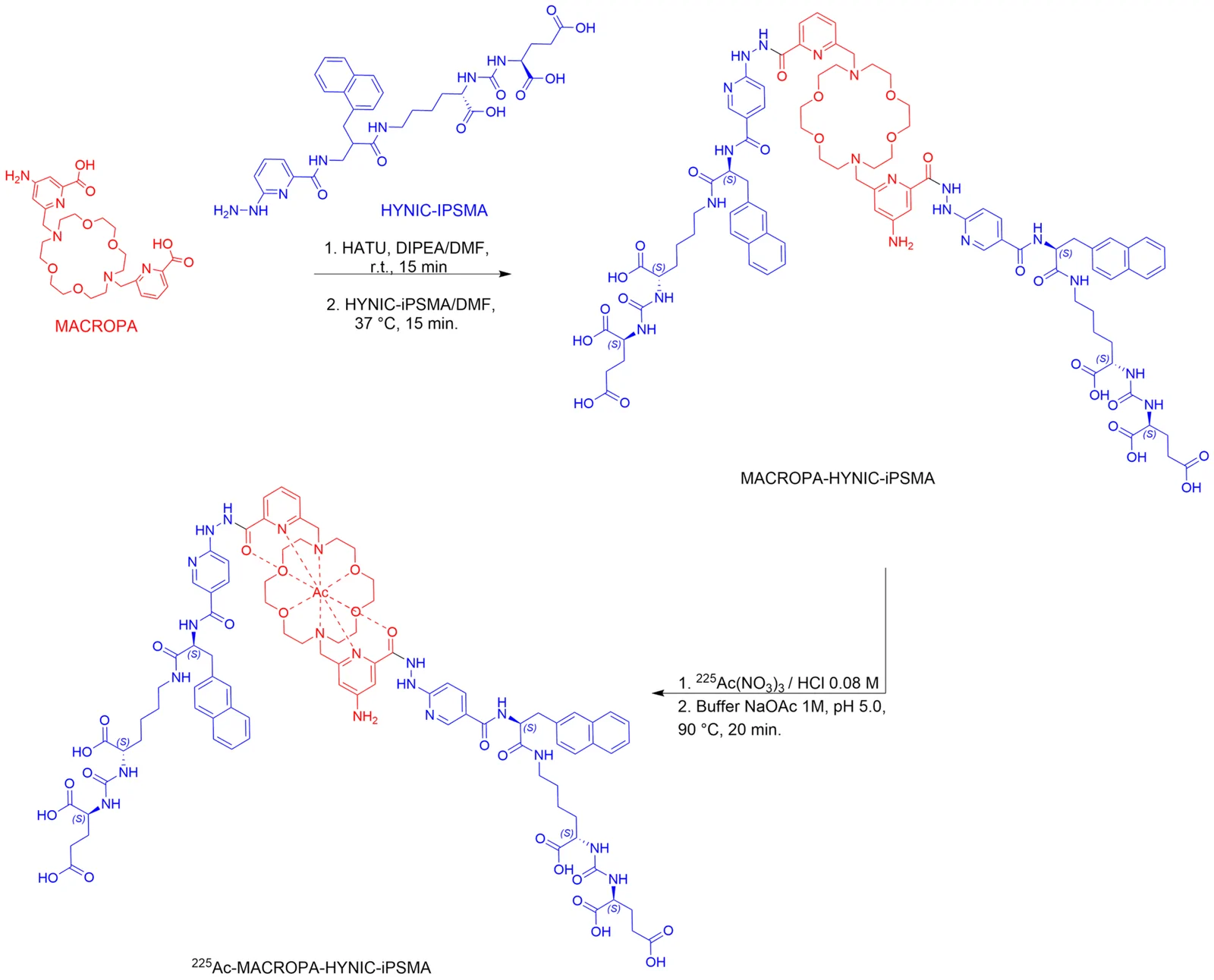
Schematic representation for the synthesis of MACROPA-iPSMA (M-iPSMA) and further radiolabeling with Ac-225.

To synthesize MACROPA-iPSMA (Figure 12), MACROPA-NH_2_ was dissolved in DMF with DIPEA and stirred at room temperature, after which HYNIC-iPSMA was added. After synthesis, the products were characterized for purity and identity using HPLC, LC-MS (Liquid Chromatography-Mass Spectrometry), NMR, FT-IR spectroscopy, and UV-Vis spectroscopy.

To activate the carboxyl groups of MACROPA for subsequent conjugation with the primary amino group of HYNIC-iPSMA, 100 µL of a solution containing 10 mg of HATU (in basic medium with DIPEA (10 µL) and DMF (200 µL)) was added to 2 mg (3.4 µmol) of MACROPA. The reaction mixture was stirred at room temperature for 15 min (solution A).

The conjugation of HYNIC-iPSMA to the activated carboxyl groups was performed by weighing 3.5 mg (5.1 μmol) of HYNIC-iPSMA and dissolving it in 200 μL of DMF—solution B.

Solution B was incorporated into solution A. The mixture was stored in a dry bath maintained at 37 °C with constant agitation for 4 h.

The purification was performed by dialysis (Tube-O-Dialyzer, Medi, 1 kDa MWCO, Biosciences, St. Louis, MO, USA), followed by additional purification by HPLC. The following protocol was followed: during the initial 1 to 3 min, 100% solvent B was maintained; between 3 and 10 min, a composition of 50% solvent B was achieved; and from 10 to 20 min, this 50% composition was maintained. The system transitioned to 70% solvent B between 20 and 23 min. At 27 min, the system stabilized at 100% solvent B until 30 min. The purified compounds were then lyophilized for subsequent characterization and radiolabeling.

#### 3.2.2. Synthesis of *p*-NCS-Bn-DOTA-iPSMA (D-iPSMA)

**Figure 13:**
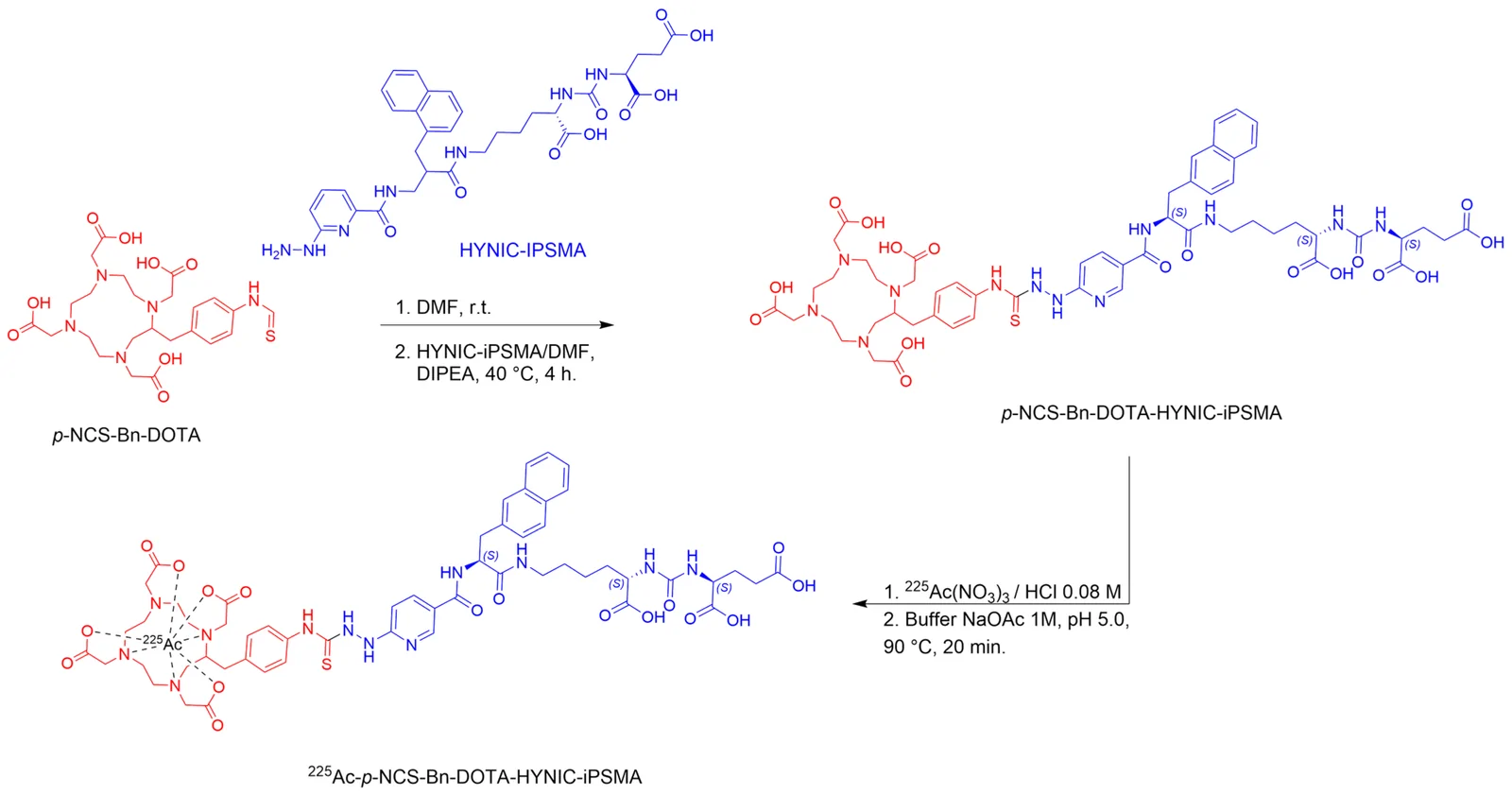
Schematic representation for the synthesis of *p*-NCS-Bn-DOTA-iPSMA (D-iPSMA) and further radiolabeling with Ac-225.

The *p*-NCS-Bn-DOTA (2 mg; 2.9 µmol) was dissolved in 200 µL of anhydrous DMF to allow the nucleophilic substitution between the isothiocyanate group (-N=C=S) on *p*-NCS-Bn-DOTA and the hydrazine group of HYNIC-iPSMA [25] (Figure 13). Then, HYNIC-iPSMA dissolved in 200 µL of DMF was added to the reaction vial, followed by 5 µL of DIPEA. The reaction was performed under basic conditions at 40 °C with an excess of HYNIC-iPSMA (6 mg, 9.2 µmol) for 4 h. The purification was performed by dialysis (Tube-O-Dialyzer, Medi, 1 KDa MWCO, Biosciences, St. Louis, MO, USA) followed by additional purification via HPLC, as indicated in Section 3.2.1.

#### 3.2.3. Chemical Characterization

After synthesis, the products were characterized for purity and identity using HPLC, LC-MS, FT-IR spectroscopy, UV-Vis spectroscopy, and NMR.

Mass spectrometry analysis (QDa Positive Scan) was performed on the samples using a UPLC/MS system equipped with a PDA detector at 280.0 nm and a Waters ACQUITY QDa mass detector operated in positive ion scan mode.

The IR spectra of M-iPSMA and D-iPSMA were acquired in solid form on an Agilent Technologies FT-IR 660 spectrometer (from 50 scans at 0.4 cm^−1^, from 400 to 4000 cm^−1^).

The UV-vis spectra (Thermo Fisher Genesys 10S spectrometer) of the M-iPSMA and DOTA-iPSMA conjugates (1 mg/mL) were obtained from 200 to 400 nm.

The ^1^H NMR spectra were recorded on a Bruker ULTRASHIELD^TM^ 500 PLUS spectrometer operating at 500 MHz in DMSO-d6 (99.5 atom % D) (Sigma-Aldrich, Milwaukee, WI, USA).

### 3.3. Radiolabeling of M-iPSMA and D-iPSMA

^225^Ac (8.5 MBq) was supplied by Van-Overeem Nuclear BV as [^225^Ac]Ac(NO_3_)_3_, which was dissolved by adding 1000 µL of 0.1 M ultrapure HCl to the original vial following the reaction: [^225^Ac]Ac(NO_3_)_3_ + 3HCl → [^225^Ac]AcCl_3_ +3HNO_3_. The vial was sterilized in an autoclave (121 °C, 15 min). Next, 200 µL aliquots of [^225^Ac]AcCl_3_ were taken and mixed with 800 µL of 1 M sodium acetate buffer (pH 5.0) to obtain a final activity of 1.7 kBq/µL. The stock solution was stored at room temperature for subsequent use.

For radiolabeling, 200 µL of the final conjugates M-iPSMA (5 × 10^−4^ M, MW = 1814 g/mol) and 100 µL of D-iPSMA (9.6 × 10^−4^ M, MW = 1203 g/mol) were added to 200 µL of [^225^Ac]AcCl_3_. The mixtures were incubated at 96 °C for 40 min. The radioligands were purified by solid-phase extraction using a Sep-Pack C-18 light cartridge (Waters, Milford, MA, USA), with the product eluted with 50% ethanol.

### 3.4. Radiochemical Purity and Stability

Radiochemical purity was determined by HPLC using a Waters system with Empower 2 Build 2154 software Empower.2 Buid2154, Waters, equipped with a UV photodiode array and a radioactivity detector. Analysis was performed using a Discovery C18 column (5 µm; 25 cm, 4.6 mm). A linear gradient of CH_3_CN—0.1% TFA/H_2_O—0.1% TFA in 30 min at a flow rate of 1 mL/min was employed. After injecting [^225^Ac]Ac-M-iPSMA or [^225^Ac]Ac-D-iPSMA, 1 mL fractions were collected to obtain free ^225^Ac and the respective purified radiopharmaceutical. Activity quantification was performed using the 218 keV gamma emission of Fr-221, assuming secular equilibrium [60]. The activity was measured using a NaI(Tl) well detector (Auto In-v-tron 4010; NML Inc., Houston, Texas, USA).

### 3.5. Radiochemical Stability in 0.9% NaCl and Human Serum Protein-Binding Assay

Purified [^225^Ac]Ac-M-iPSMA and [^225^Ac]Ac-D-iPSMA were incubated at 37 °C in 0.9% NaCl solution (1:1 *v*/*v*) to evaluate radiochemical stability over 10 days. Human serum samples were incubated at a 1:1 (*v*/*v*) ratio, proteins were precipitated by adding acetonitrile at a 1:1 (*v*/*v*) ratio, followed by vortexing and centrifugation at 10,000× *g* rpm for 10 min. Radioactivity in the pellet and supernatant fractions was measured to calculate the percentage of protein-bound radiopharmaceutical.

### 3.6. Cell Culture

Cell lines, 4T1 (CRL-2539) PSMA-positive [34], HCT116 (CCL-247EMT, Human Colorectal Carcinoma) PSMA-positive [61], and AR42J (CLR-1492) PSMA-negative [62,63], were obtained from the American Type Culture Collection (Manassas, VA, USA). Cells were used at low passage numbers after thawing and were routinely monitored for mycoplasma contamination. PSMA expression under the experimental conditions was further confirmed by immunofluorescence and Western blot. These cells were grown in Roswell Park Memorial Institute 1640 (RPMI-1640) medium supplemented with 10% fetal bovine serum (FBS) and 100 U/mL penicillin and streptomycin at 37 °C in a 5% CO_2_ atmosphere.

### 3.7. PSMA Expression

PSMA expression was evaluated by immunofluorescence and Western blot (WB). For immunofluorescence, cells were fixed with 4% paraformaldehyde for 20 min, permeabilized with 0.5% TritonX-100, and blocked with 1% bovine serum albumin. The cells were then incubated overnight with anti-PSMA antibody (Abcam Cat. ab268061) at a dilution of 1:100. The cells were then incubated for 1 h with Alexa Fluor 594-conjugated goat anti-rabbit IgG (H + L) (Invitrogen Cat. A32731). DAPI was used for nuclear staining, and an Agilent BioTek Cytation C10 Confocal Imaging Reader was used for fluorescence images.

For WB, the cells were lysed and 30 μg protein per sample was resolved by polyacrylamide gel electrophoresis, transferred to PVDF membranes (Merck Millipore) and blocked for 1 h at rt with BSA 5% (bovine serum albumin, Sigma-Aldrich) and probed with anti-PSMA (Abcam Cat. ab268061, dilution 1:1000) and anti-Actin (Sigma-Aldrich Cat. A3854, dilution 1:20,000). The primary antibodies were incubated overnight at 4 °C, then washed. Species-specific HRP-conjugated secondary antibodies were incubated for 1 h at rt and washed. Finally, the membranes were incubated with Super Signal West Femto substrate (Thermo Fisher Scientific, Waltham, MA, USA), and signals were detected using the Xtreme in vivo imaging system (Bruker, Billerica, MA, USA).

### 3.8. Uptake and Internalization

The [^225^Ac]Ac-M-iPSMA and [^225^Ac]Ac-D-iPSMA uptake was performed on HCT116, 4T1 PSMA-positive cells, and AR42J PSMA-negative cells (low expression considered as the baseline for the negative control). The uptake and internalization protocol was based on previously reported PSMA-targeted radiopharmaceutical methods using sequential washing and acid-stripping steps to distinguish total cell-associated activity from internalized activity [25,26,31]. Each cell line was counted to 5 × 10^5^ cells in 0.5 mL of PBS in Eppendorf tubes and subsequently incubated at 37 °C with 30 µL (110 kBq) of [^225^Ac]Ac-M-iPSMA and [^225^Ac]Ac-D-iPSMA. At 1 h, the tubes were centrifuged at 3000× *g* rpm for 5 min, followed by two PBS washes. The radioactivity associated with the pellet at this step represents the uptake and internalization.

To facilitate the surface-bound radioligand removal of each ^225^Ac-radiopharmaceutical, 500 µL of 0.2 M CH_3_COOH/0.5 M NaCl was added after the pellet was resuspended, centrifuged, and rinsed with PBS. The pellet was measured to register the [^225^Ac]Ac-M-iPSMA or [^225^Ac]Ac-D-iPSMA internalization in each cell line.

To validate specificity, the 4T1 cell line (5 × 10^5^ cells in 0.5 mL) with the highest PSMA expression was preincubated with a 1 mM HYNIC-iPSMA solution to block receptors. Then, 110 kBq [^225^Ac]Ac-M-iPSMA was incubated and uptake was measured after 1 h, as previously described.

### 3.9. Cell Binding

Because Ac ([Rn]6d^1^7s^2^) closely resembles La ([Xe]5d^1^6s^2^) in electronic configuration and trivalent oxidation state, La(III) has been widely employed as a chemical surrogate for Ac-225 [64,65,66]. A saturation binding assay was used to determine the affinity of purified La-M-iPSMA and La-D-iPSMA. A 4T1 cellular suspension at 1 × 10^6^ cells was inoculated into 6-well plates and cultured for 24 h at 37 °C, 5% CO_2_, and 85% humidity to promote adherence. The cells were then cooled on ice for 30 min. The cells were incubated with seven distinct concentrations (n = 3) of La-M-iPSMA and La-D-iPSMA (ranging from 200 nM to 0.25 nM in 200 µL) for 2 h at 4 °C. Simultaneously, non-specific binding was evaluated using “cold peptide” (iPSMA, 1 mM). Subsequently, quantitative determination of lanthanum was performed by inductively coupled plasma quadrupole mass spectrometry (ICP-QMS) using an Agilent 7700X instrument in the free (supernatant) and bound (membrane-bound and internalized) radiopharmaceutical fractions. Previously prepared standard solutions representing 100% of the initial concentration for each treatment condition were used. The specific binding was calculated as the difference between total binding and non-specific binding. The dissociation constant (K_d_) and maximum binding capacity (B_Zn_) were determined by nonlinear regression analysis using OriginLab software OriginPro, Version 2024, 10.1.0.178 (OriginLab Corporation; Northampton, MA, USA).

### 3.10. Apoptotic Cell Death

Apoptosis in the AR42J, HCT116, and 4T1 cell lines was evaluated using the Muse™ Caspase-3/7 kit (Cat. No. MCH100108, Merck Millipore; Burlington, MA, USA). Briefly, cells were seeded in cell culture flasks at a density of 1 × 10^6^ cells and exposed to 2–6 Gy doses of purified [^225^Ac]Ac-D-iPSMA and [^225^Ac]Ac-M-iPSMA. Untreated cells were used as the control group. First, cells were trypsinized and suspended in 50 μL of 1X Assay Buffer BA. Subsequently, 5 μL of the working solution of Caspase-3/7 reagent was added to each cell suspension. The suspension was then gently vortexed and incubated at 37 °C with 5% CO_2_ for 30 min. After incubation, 150 μL of Caspase 7-AAD working solution was added to each tube. Finally, tubes were incubated for 5 min, protected from light. Fluorescence was measured by flow cytometry using a Muse Cell Analyzer (Merck Millipore; Burlington, MA, USA) in triplicate.

### 3.11. Clonogenic Assay

4T1, HCT116, and AR42J cells were exposed to 1, 2, and 3 Gy doses of [^225^Ac]Ac-D-iPSMA or [^225^Ac]Ac-M-iPSMA (n = 3). After removal of excess radiopharmaceutical, cells were resuspended in fresh medium and seeded at 3 × 10^3^ cells per well in six-well plates containing 2 mL RPMI medium. Cultures were incubated for 7 days until visible colonies formed. Colonies were fixed with 4.5% paraformaldehyde for 30 min, stained with 5% crystal violet for 30 min, rinsed with distilled water, photographed, and counted using ImageJ v1.54t. The surviving fraction was calculated from colony counts normalized to untreated controls and plating efficiency.

### 3.12. DNA Damage

For the evaluation of DNA damage, the phosphorylation of the Ser-139 gamma histone variant H2A.X and the phosphorylation of the Ser-1981 of Ataxia Telangiectasia Mutated (ATM) were used as biomarkers with the Muse™ Multi-color DNA Damage kit, Cat. No. MCH200107 (Merck Millipore; Burlington, MA, USA). Briefly, cells were seeded in cell culture flasks at a density of 1 × 10^6^ cells and exposed to 2 to 6 Gy radiation doses of [^225^Ac]Ac-D-iPSMA and [^225^Ac]Ac-M-iPSMA. Untreated cells were used as the control group. First, cells were trypsinized and suspended in 500 μL of 1X Assay Buffer. The cells were then fixed and permeabilized. The suspension was rinsed and incubated with the antibody working cocktail solution for 30 min in the dark at room temperature. After incubation, 200 μL of 1X assay buffer was added to each tube. Fluorescence was measured by flow cytometry using a Muse Cell Analyzer (Merck Millipore; Burlington, MA, USA) and was conducted in triplicate.

### 3.13. Human Phospho-Kinase Array

To understand how cells respond to radiopharmaceutical treatment, we analyzed the phosphorylation profiles of kinases and their protein substrates. Briefly, 4T1 Breast cancer cells were rinsed with cold PBS and lysed in buffer supplemented with cOmplete^TM^ EDTA-free Ultra Protease Inhibitor Cocktail (Sigma-Aldrich) and 1X PhosSTOP^TM^ (Sigma-Aldrich) at 4 °C for 30 min. Following centrifugation at 14,000× *g* for 5 min, the supernatants were transferred to a clean tube, and protein concentrations were determined using the Precision Red Advanced Protein Assay (Cytoskeleton, Inc., ADV02-A; Denver, CO, USA). Lysates were diluted and analyzed using the Human Phospho-Kinase Array (Proteome Profiler; R&D Systems; Minneapolis, MN, USA) according to the manufacturer’s instructions. Membranes were scanned using the in vivo Xtreme imaging system (Bruker, Billerica, MA, USA).

### 3.14. ROS Production and Fluorescence Localization Assay

ROS production was evaluated in 4T1 cells after exposure to [^177^Lu]Lu-D-iPSMA, [^225^Ac]Ac-M-iPSMA, or their combined treatment. Cells were incubated with the fluorescent ROS probe according to the manufacturer’s instructions, washed to remove excess probe, and imaged using the fluorescence microscopy settings applied consistently across treatment groups. Fluorescence intensity was used as a comparative indicator of intracellular ROS accumulation, and cellular localization was interpreted descriptively from the fluorescence distribution within the imaged cells. The assay was used to compare relative oxidative stress among treatment groups and was not designed to prove bystander signaling or a specific subcellular mechanism.

### 3.15. Tumor Induction in Mice

Tumor development was initiated by subcutaneously injecting 1 × 10^6^ 4T1 cells into 6-week-old female Balb-c mice obtained from the Laboratory Animal Production and Experimentation Unit (UPEAL) of CINVESTAV. Tumor growth was monitored by visual inspection.

### 3.16. Biodistribution and Dosimetry Estimation

In vivo studies were performed in 4T1 tumor-bearing Balb-c (tumor size 150–250 mm^3^). [^225^Ac]Ac-M-iPSMA was chosen to perform in vivo studies. In total, 50 uL of a 1 mg/mL solution (0.543 mM of M-iPSMA) was intravenously injected into the tail vein (0.074 MBq, specific activity 313 nmol/MBq). For biodistribution studies, mice were euthanized at 0.5, 1,2, 3, 24, and 48 h (n = 3) by CO_2_ asphyxiation, and the organs and tissues of interest were removed and counted at a NaI(Tl) gamma counter. The cpm from all samples were corrected for decay and background and converted to %ID/g by comparison with a standard.

The internal dosimetry was performed following the MIRD (Medical Internal Radiation Dose) formalism. An activity of 0.074 MBq of [^225^Ac]Ac-PSMA was administered to 25 g mice. The temporal biodistribution data were fitted using multi-exponential pharmacokinetic models: a tri-exponential model for the blood, kidneys, spleen, bone, liver, and tumor. The S-factors per kidney, spleen, bone, and liver were the sum of the contribution of decay energy of Ac-225.

The S factor for tumor self-irradiation was estimated using the unit-density sphere model. The Time-Integrated Activity Coefficient (TIAC) was calculated by integrating the exponential functions from t = 0 to t = ∞. The S factor for tumor self-irradiation was estimated from data on the decay energy of ^225^Ac and its decay chain (^221^Fr, ^213^Bi, ^213^Po, ^209^Tl, ^209^Pb) published at https://www.nndc.bnl.gov/ (accessed on 12 July 2026), with a total energy of 28.30 MeV per decay, considering alpha particles, conversion electrons, and Auger electrons, according to the recommendations of MIRD Pamphlet No. 22 for alpha emitters [10]. For a spherical tumor weighing 0.15 g (approximate radius of 3.3 mm), complete absorption of the emitted energy was assumed due to the short range of alpha particles (50–100 μm in tissue), resulting in an S factor of 3.02 × 10^−8^ Gy/(Bq·s).

The absorbed dose was calculated using the MIRD formalism. For the in vitro experiments, the administered activities were converted into estimated absorbed doses by considering the initial activity added to each cell suspension, the exposure time, and radioactive decay during incubation. Complete local energy deposition of alpha particles was assumed, together with a uniform distribution of activity within the cell population and a negligible contribution from adjacent wells. This assumption is justified by the alpha-particle range (~50–80 μm), which exceeds typical cell dimensions (10–20 μm). The contribution of ^225^Ac and its progeny was calculated using the decay-chain energy deposition approach commonly applied in alpha-emitter dosimetry. The reported dose values (Gy) therefore correspond to estimated absorbed doses under the defined experimental geometry and incubation conditions.

## 4. Conclusions

The study describes the design, synthesis, and preclinical evaluation of two new ^225^Ac-labeled PSMA-targeting radiopharmaceuticals: the monomeric [^225^Ac]Ac-D-iPSMA (using a DOTA chelator) and the dimeric [^225^Ac]Ac-M-iPSMA (using a MACROPA chelator). Both are based on the HYNIC-iPSMA pharmacophore, which has already been proven in a clinical setting. A multi-level computational approach was used, including ensemble docking with deep-learning scoring (using GNINA 1.1), extended molecular dynamics simulations, and well-tempered metadynamics. This approach guided the selection of the chelator and scaffold before synthesis, providing a structural and thermodynamic basis for the binding characteristics. Two radiopharmaceuticals were obtained with high radiochemical purity. La(III) saturation binding assays showed nanomolar affinity for PSMA, consistent with predictions.

The study provides the first preclinical evaluation of HYNIC-iPSMA-based ^225^Ac radiopharmaceuticals in triple-negative breast cancer (4T1) and colorectal cancer (HCT116) models, which is a meaningful contribution to the field of PSMA-targeted therapy beyond prostate cancer. In vitro studies showed that [^225^Ac]Ac-D-iPSMA had greater cytotoxic potency. [^225^Ac]Ac-M-iPSMA showed better radiochemical stability under physiological conditions, due to the thermodynamic stability of the MACROPA macrocycle. It also produced a favorable tumor-to-kidney absorbed dose ratio of 54:1 in BALB/c mice bearing 4T1 tumors, a dosimetric factor important for clinical translation. Moreover, combining [^177^Lu]Lu-D-iPSMA with [^225^Ac]Ac-M-iPSMA increased reactive oxygen species generation in 4T1 cells compared to either agent alone. It provides a basis for tandem alpha–beta radionuclide therapy using the complementary radiobiological properties of high-LET alpha particles and the crossfire effect of beta emitters.

The data establish [^225^Ac]Ac-D-iPSMA and [^225^Ac]Ac-M-iPSMA as radiopharmaceutical candidates with favorable preclinical profiles for PSMA-targeted alpha therapy in non-prostate solid tumors. However, in vivo therapeutic efficacy remains to be demonstrated. In vivo biodistribution and dosimetry were obtained for [^225^Ac]Ac-M-iPSMA; the dataset for [^225^Ac]Ac-D-iPSMA could not be completed due to logistical limitations, a recognized limitation. Even with these gaps, the in vitro data across two clinically relevant cancer models, combined with the favorable dosimetric profile of [^225^Ac]Ac-M-iPSMA in vivo, provide a substantive preclinical basis for progressing both compounds into therapeutic efficacy and toxicity studies.

A practical consideration for clinical translation of ^225^Ac-based radiopharmaceuticals is the currently constrained global supply. This limitation is recognized across the targeted alpha therapy field and is not specific to the HYNIC-iPSMA platform. Subsequent studies will prioritize in vivo therapeutic efficacy for both [^225^Ac]Ac-D-iPSMA and [^225^Ac]Ac-M-iPSMA, full comparative biodistribution and dosimetric characterization of [^225^Ac]Ac-D-iPSMA, and formal evaluation of the tandem [^177^Lu]Lu-iPSMA/[^225^Ac]Ac-iPSMA combination therapy in vivo.

## Figures and Tables

**Table 1:** Molecular Docking. Docking scores and key interactions of the evaluated radiopharmaceuticals at the PSMA receptor.

Ligand	CNN Docking Score (kcal/mol)	Key Residue Involved	Dominant Interactions
MACROPA-HYNIC-iPSMA	−16.94 ± 0.72	PHE209, ARG463, TYR471, SER503, ILE512, ARG534, TRP541, LYS610, TYR692, TYR700	H-bond, π-cation, π–π stacking
*p*-NCS-Bn-DOTA-iPSMA	−13.43 ± 0.45	PHE209, ARG210, LEU261, ARG463, ARG534, ARG536, TYR700	H-bond, π-cation, π–π stacking
HYNIC-iPSMA	−12.08 ± 0.32	LYS207, GLY427, ARG534, LYS539, TYR552, TYR700, Zn815, Zn816	H-bond, π-cation, π–π stacking, salt bridge, metal coordination

**Table 2:** Free energy profile derived from well-tempered metadynamics simulations for selected iPSMA bound to the PSMA.

Ligands	Bound State *	Unbound State *	ΔGbind *	* ΔΔG
M-iPSMA	−37.6 ± 1.92	−20.62 ± 2.83	−16.97	358.46
D-iPSMA	−48.52 ± 4.02	−15.31 ± 1.77	−33.21	342.22
HYNIC-iPSMA	−784.63 ± 9.39	−153.65 ± 46.83	−630.98	−255.55

* All units are expressed as kcal/mol and averaged from triplicate simulations.

**Table 3:** Total nuclear transformations (N) and radiation absorbed doses in target organs of 4T1-bearing BALB/c mice produced by [^225^Ac]Ac-M-iPSMA.

Organ	Radiopharmacokinetic Model	$N=\int_{t=0}^{t=\infty \;d}A\left(t\right)\mathrm{dt}$ MBq.s	Radiation Absorbed Dose Gy/0.074 MBq
Blood	$\mathrm{Ah}\left(t\right)=1.33e^{-41.3029t}+7.32e^{-0.7329t}$	10.02	_
Liver	$\mathrm{Ah}\left(t\right)=24.9e^{-10.5029t}+3.06e^{-0.0338t}+0.186e^{-0.0337t}$	241.92	3.17
Tumor	$\mathrm{Ah}\left(t\right)=35.9e^{-11.3029t}+104e^{-0.10029t}+0.25e^{-0.0029t}$	2929	88.17
Kidney	$\mathrm{Ah}\left(t\right)=-51.6e^{-1.4929t}+40.6e^{-0.9529t}+0.412e^{-0.0029t}$	21.42	1.62
Bone	$\mathrm{Ah}\left(t\right)=0.0238e^{-10.203t}+1.78e^{-4.123t}+0.145e^{-0.0323t}$	13.11	0.108
Spleen	$\mathrm{Ah}\left(t\right)=63.9e^{-12.6029t}+0.385e^{-0.06881t}+0.176e^{-0.06881t}$	35.23	7.19

## Data Availability

The original contributions presented in this study are included in the article/Supplementary Materials. Further inquiries can be directed to the corresponding authors.

## Supplementary Materials

The following supporting information can be downloaded at: https://www.mdpi.com/article/10.3390/ijms27177785/s1.

## Abbreviations

The following abbreviations are used in this manuscript:

ATM	Ataxia Telangiectasia Mutated
Bmax	Maximum Binding Capacity
COM	Center of Mass
DAPI	4′,6-Diamidino-2-Phenylindole
DIPEA	N,N-Diisopropylethylamine
DMF	Dimethylformamide
DOTA	1,4,7,10-Tetraazacyclododecane-1,4,7,10-Tetraacetic Acid
DSB	Double-Strand Break
EGFR	Epidermal Growth Factor Receptor
FESs	free energy surfaces
FT-IR	Fourier Transform Infrared Spectroscopy
GNINA	Graph Neural Network-based Docking Software
GSK-3	Glycogen Synthase Kinase-3
γ-H2AX	Phosphorylated histone
HER2	Human Epidermal Growth Factor Receptor 2
^1^H NMR	Proton Nuclear Magnetic Resonance Spectroscopy
HPLC	High-Performance Liquid Chromatography
HRP	Horseradish Peroxidase
HYNIC	Hydrazinonicotinamide
ICP-QMS	Inductively Coupled Plasma Quadrupole Mass Spectrometry
Kd	Equilibrium dissociation constant
LET	Linear Energy Transfer
MACROPA	N,N′-bis[(6-carboxypyridin-2-yl)methyl]-4,13-diaza-18-crown-6
MAPK	Mitogen-Activated Protein Kinase
mCRPC	Metastatic Castration-Resistant Prostate Cancer
MIRD	Medical Internal Radiation Dose
PSA50	Prostate-Specific Antigen 50% response
RMSD	root-mean-square deviation
RMSF	root-mean-square Fluctuation
WNK1	With No Lysine Kinase 1
wt-MetaD	Well-Tempered Metadynamics

## References

[1] Ghosh A., Heston W.D. (2004). Tumor target prostate specific membrane antigen (PSMA) and its regulation in prostate cancer. J. Cell. Biochem..

[2] Chang S.S., Reuter V.E., Heston W.D., Bander N.H., Grauer L.S., Gaudin P.B. (1999). Five different anti-prostate-specific membrane antigen (PSMA) antibodies confirm PSMA expression in tumor-associated neovasculature. Cancer Res..

[3] Haffner M.C., Kronberger I.E., Ross J.S., Sheehan C.E., Zitt M., Mühlmann G., Öfner D., Zelger B., Ensinger C., Yang X.J. (2009). Prostate-specific membrane antigen expression in the neovasculature of gastric and colorectal cancers. Hum. Pathol..

[4] Sartor O., de Bono J., Chi K.N., Fizazi K., Herrmann K., Rahbar K., Tagawa S.T., Nordquist L.T., Vaishampayan N., El-Haddad G. (2021). Lutetium-177-PSMA-617 for Metastatic Castration-Resistant Prostate Cancer. N. Engl. J. Med..

[5] Fendler W.P., Rahbar K., Herrmann K., Kratochwil C., Eiber M. (2017). ^177^Lu-PSMA radioligand therapy for prostate cancer. J. Nucl. Med..

[6] Luna-Gutiérrez M., Hernandez-Ramirez R., Soto-Abundiz A., García-Pérez O., Ancira-Cortez A., López-Buenrostro S., Gibbens-Bandala B., Soldevilla-Gallardo I., Lara-Almazán N., Rojas-Perez M. (2023). Improving overall survival and quality of life in patients with prostate cancer and neuroendocrine tumors using ^177^Lu-iPSMA and ^177^Lu-DOTATOC: Experience after 905 treatment doses. Pharmaceutics.

[7] Buteau J.P., Kostos L., Jackson P.A., Xie J., Haskali M.B., Alipour R., McIntosh L.E., Emmerson B., MacFarlane L., Martin C.A. (2025). First-in-human results of terbium-161 [^161^Tb] Tb-PSMA-I&T dual beta–Auger radioligand therapy in patients with metastatic castration-resistant prostate cancer (VIOLET): A single-centre, single-arm, phase 1/2 study. Lancet Oncol..

[8] Raychaudhuri R., Mo G., Tuchayi A.M., Graham L., Gulati R., Pritchard C.C., Haffner M.C., Yezefski T., Hawley J.E., Cheng H.H. (2024). Genomic correlates of prostate-specific membrane antigen expression and response to ^177^Lu-PSMA-617: A retrospective multicenter cohort study. JCO Precis. Oncol..

[9] Sartor O., Ledet E., Huang M., Schwartz J., Lieberman A., Lewis B., Layton J., Barata P., Jang A., Hawkins M. (2023). Prediction of resistance to ^177^Lu-PSMA therapy by assessment of baseline circulating tumor DNA biomarkers. J. Nucl. Med..

[10] Sgouros G., Roeske J.C., McDevitt M.R., Palm S., Allen B.J., Fisher D.R., Brill A.B., Song H., Howell R.W., Akabani G. (2010). MIRD Pamphlet No. 22 (abridged): Radiobiology and dosimetry of alpha-particle emitters for targeted radionuclide therapy. J. Nucl. Med..

[11] Cruz-Nova P., Trujillo-Nolasco M., Aranda-Lara L., Ferro-Flores G., Ocampo-García B. (2025). Radiobiological effect of alpha particles. The scientific basis of targeted alpha-particle therapy. Nucl. Med. Biol..

[12] Thiele N.A., Wilson J.J. (2018). Actinium-225 for Targeted α Therapy: Coordination Chemistry and Current Chelation Approaches. Cancer Biother. Radiopharm..

[13] Kratochwil C., Bruchertseifer F., Rathke H., Bronzel M., Apostolidis C., Weichert W., Haberkorn U., Giesel F.L., Morgenstern A. (2017). Targeted α-Therapy of Metastatic Castration-Resistant Prostate Cancer with ^225^Ac-PSMA-617: Dosimetry Estimate and Empiric Dose Finding. J. Nucl. Med..

[14] Satapathy S., Sood A., Das C.K., Mittal B.R. (2021). Evolving role of ^225^Ac-PSMA radioligand therapy in metastatic castration-resistant prostate cancer—A systematic review and meta-analysis. Prostate Cancer Prostatic Dis..

[15] Kairemo K., Kgatle M., Bruchertseifer F., Morgernstern A., Sathekge M.M. (2024). Design of ^225^Ac-PSMA for targeted alpha therapy in prostate cancer. Ann. Transl. Med..

[16] Kratochwil C., Haberkorn U., Giesel F.L. (2020). ^225^Ac-PSMA-617 for Therapy of Prostate Cancer. Semin. Nucl. Med..

[17] Kannengießer S. (2013). Optimization of the Synthesis of Ac-225-labelled DOTA-Radioimmunoconjugates for Targeted Alpha Therapy, based on Investigations on the Complexation of Trivalent Actinides by DOTA.

[18] Matazova E.V., Egorova B.V., Zubenko A.D., Pashanova A.V., Mitrofanov A.A., Fedorova O.A., Ermolaev S.V., Vasiliev A.N., Kalmykov S.N. (2023). Insights into actinium complexes with tetraacetates─AcBATA versus AcDOTA: Thermodynamic, structural, and labeling properties. Inorg. Chem..

[19] Cheveau M., Moreau M., Grohmann A., Robert S., Cochet A., Collin B., Boschetti F., Poty S., Denat F. (2025). Versatile bifunctional PYTA derivatives for actinium-225 radiolabeling: A comparison to gold standards. ChemRxiv.

[20] Thiele N.A., Brown V., Kelly J.M., Amor-Coarasa A., Jermilova U., MacMillan S.N., Nikolopoulou A., Ponnala S., Ramogida C.F., Robertson A.K. (2017). An eighteen-membered macrocyclic ligand for actinium-225 targeted alpha therapy. Angew. Chem. Int. Ed..

[21] Reissig F., Zarschler K., Novy Z., Petrik M., Bendova K., Kurfurstova D., Bouchal J., Ludik M.-C., Brandt F., Kopka K. (2022). Modulating the pharmacokinetic profile of Actinium-225-labeled macropa-derived radioconjugates by dual targeting of PSMA and albumin. Theranostics.

[22] Banerjee S.R., Pullambhatla M., Byun Y., Nimmagadda S., Green G., Fox J.J., Horti A., Mease R.C., Pomper M.G. (2010). 68Ga-labeled inhibitors of prostate-specific membrane antigen (PSMA) for imaging prostate cancer. J. Med. Chem..

[23] García-Pérez F.O., Davanzo J., López-Buenrostro S., Santos-Cuevas C., Ferro-Flores G., Jímenez-Ríos M.A., Scavuzzo A., Santana-Ríos Z., Medina-Ornelas S. (2018). Head to head comparison performance of ^99m^Tc-EDDA/HYNIC-iPSMA SPECT/CT and ^68^Ga-PSMA-11 PET/CT a prospective study in biochemical recurrence prostate cancer patients. Am. J. Nucl. Med. Mol. Imaging.

[24] Tually P., Quinto V.G., Omar Y., Novruzov F., Yudistiro R., Sathekge M., Currie G., Galette P., Patel N., Brown T. (2024). Real world experience with [^99m^Tc] Tc-HYNIC-iPSMA SPECT prostate cancer detection: Interim results from the global NOBLE registry. EJNMMI Rep..

[25] Ferro-Flores G., Luna-Gutiérrez M., Ocampo-García B., Santos-Cuevas C., Azorín-Vega E., Jiménez-Mancilla N., Orocio-Rodríguez E., Davanzo J., García-Pérez F.O. (2017). Clinical translation of a PSMA inhibitor for ^99m^Tc-based SPECT. Nucl. Med. Biol..

[26] Santos-Cuevas C., Davanzo J., Ferro-Flores G., García-Pérez F.O., Ocampo-García B., Ignacio-Alvarez E., Gómez-Argumosa E., Pedraza-López M. (2017). ^99m^Tc-labeled PSMA inhibitor: Biokinetics and radiation dosimetry in healthy subjects and imaging of prostate cancer tumors in patients. Nucl. Med. Biol..

[27] Flores G.F., García B.E.O., Gutiérrez M.A.L., Cuevas C.L.S., Vega E.P.A., Mancilla N.P.J., Jiménez T.H. (2024). 177Lu-DOTA-HYNIC-iPSMA as a Therapeutic Radiopharmaceutical Targeting Prostate-Specific Membrane Antigen. U.S. Patent.

[28] Santos-Cuevas C., Ferro-Flores G., García-Pérez F.O., Jiménez-Mancilla N., Ramírez-Nava G., Ocampo-García B., Luna-Gutiérrez M., Azorín-Vega E., Davanzo J., Soldevilla-Gallardo I. (2018). ^177^Lu-DOTA-HYNIC-Lys(Nal)-Urea-Glu: Biokinetics, Dosimetry, and Evaluation in Patients with Advanced Prostate Cancer. Contrast Media Mol. Imaging.

[29] Hooijman E.L., de Jong J.R., Ntihabose C.M., Bruchertseifer F., Morgenstern A., Seimbille Y., Brabander T., Koolen S.L., de Blois E. (2025). Ac-225 radiochemistry through the lens of [^225^Ac] Ac-DOTA-TATE. EJNMMI Radiopharm. Chem..

[30] Hooijman E.L., Chalashkan Y., Ling S.W., Kahyargil F.F., Segbers M., Bruchertseifer F., Morgenstern A., Seimbille Y., Koolen S.L., Brabander T. (2021). Development of [^225^Ac] Ac-PSMA-I&T for targeted alpha therapy according to GMP guidelines for treatment of mCRPC. Pharmaceutics.

[31] Liu S., Ellars C.E., Edwards D.S. (2003). Ascorbic acid: Useful as a buffer agent and radiolytic stabilizer for metalloradiopharmaceuticals. Bioconjug. Chem..

[32] Basken N.E., Mathias C.J., Lipka A.E., Green M.A. (2008). Species dependence of [^64^Cu] Cu-Bis (thiosemicarbazone) radiopharmaceutical binding to serum albumins. Nucl. Med. Biol..

[33] Zitzmann-Kolbe S., Papple A., Poethko T., Moen I., Schatz C., Hillier S., Babich J.W., Cuthbertson A., Hagemann U.B. (2024). Preclinical evaluation of an actinium-225 labeled PSMA-targeting small molecule (^225^Ac-PSMA-Trillium (BAY 3563254)) for the treatment of metastatic castration resistant prostate cancer (mCRPC). Cancer Res..

[34] Shirke A.A., Wang J., Ramamurthy G., Mahanty A., Walker E., Zhang L., Panigrahi A., Wang X., Basilion J.P. (2024). Prostate Specific Membrane Antigen Expression in a Syngeneic Breast Cancer Mouse Model. Mol. Imaging Biol..

[35] O’Keefe D.S., Bacich D.J., Huang S.S., Heston W.D.W. (2018). A Perspective on the Evolving Story of PSMA Biology, PSMA-Based Imaging, and Endoradiotherapeutic Strategies. J. Nucl. Med..

[36] Wichmann C.W., Morgan K.A., Cao Z., Osellame L.D., Guo N., Gan H., Reilly E., Burvenich I.J., O’Keefe G.J., Donnelly P.S. (2024). Radiolabeling and preclinical evaluation of therapeutic efficacy of ^225^Ac-ch806 in glioblastoma and colorectal cancer xenograft models. J. Nucl. Med..

[37] Xie L., Qin J., Song C., Yin J., Wu R., Chen H., Dong Y., Wang N., Chen L., Hong B. (2025). ^157^Gd-DOTA-PSMA as theranostic bio-gadolinium agent for prostate cancer targeted gadolinium neutron capture therapy. J. Cancer Res. Clin. Oncol..

[38] McCubrey J.A., Steelman L.S., Bertrand F.E., Davis N.M., Sokolosky M., Abrams S.L., Montalto G., D’Assoro A.B., Libra M., Nicoletti F. (2014). GSK-3 as potential target for therapeutic intervention in cancer. Oncotarget.

[39] Xiu M., Li L., Li Y., Gao Y. (2022). An update regarding the role of WNK kinases in cancer. Cell Death Dis..

[40] Medina M.A., Oza G., Sharma A., Arriaga L.G., Hernández Hernández J.M., Rotello V.M., Ramirez J.T. (2020). Triple-Negative Breast Cancer: A Review of Conventional and Advanced Therapeutic Strategies. Int. J. Environ. Res. Public Health.

[41] Tang Y., Zhou Y., Fan S., Wen Q. (2022). The multiple roles and therapeutic potential of HSP60 in cancer. Biochem. Pharmacol..

[42] Benešová M., Bauder-Wüst U., Schäfer M., Klika K.D., Mier W., Haberkorn U., Kopka K., Eder M. (2016). Linker Modification Strategies to Control the Prostate-Specific Membrane Antigen (PSMA)-Targeting and Pharmacokinetic Properties of DOTA-Conjugated PSMA Inhibitors. J. Med. Chem..

[43] Luhung A., Hasanah A.N., Ramdhani D. (2026). Radiopharmaceutical: An Update and Comparison of Preclinical Investigation Result of Alpha and Beta Emitter Radioisotope. Drug Des. Dev. Ther..

[44] AM Engbers P., Nonnekens J., Sabatella M. (2026). Cellular responses to targeted radionuclide therapy: Rethinking radiobiology under continuous low dose rates. Front. Oncol..

[45] Veach D.R., Storey C.M., Lückerath K., Braun K., von Bodman C., Lamminmäki U., Kalidindi T., Strand S.E., Strand J., Altai M. (2021). PSA-Targeted Alpha-, Beta-, and Positron-Emitting Immunotheranostics in Murine Prostate Cancer Models and Nonhuman Primates. Clin. Cancer Res..

[46] Wernicke A.G., Varma S., Greenwood E.A., Christos P.J., Chao K.C., Liu H., Bander N.H., Shin S.J. (2014). Prostate-specific membrane antigen expression in tumor-associated vasculature of breast cancers. Apmis.

[47] Luining W.I., Cysouw M.C.F., Meijer D., Hendrikse N.H., Boellaard R., Vis A.N., Oprea-Lager D.E. (2022). Targeting PSMA Revolutionizes the Role of Nuclear Medicine in Diagnosis and Treatment of Prostate Cancer. Cancers.

[48] Rosar F., Krause J., Bartholomä M., Maus S., Stemler T., Hierlmeier I., Linxweiler J., Ezziddin S., Khreish F. (2021). Efficacy and Safety of [^225^Ac]Ac-PSMA-617 Augmented [^177^Lu]Lu-PSMA-617 Radioligand Therapy in Patients with Highly Advanced mCRPC with Poor Prognosis. Pharmaceutics.

[49] Ling S.W., de Blois E., Hooijman E., van der Veldt A., Brabander T. (2022). Advances in 177Lu-PSMA and 225Ac-PSMA Radionuclide Therapy for Metastatic Castration-Resistant Prostate Cancer. Pharmaceutics.

[50] An S., Huang G., Liu J., Wei W. (2022). PSMA-targeted theranostics of solid tumors: Applications beyond prostate cancers. Eur. J. Nucl. Med. Mol. Imaging.

[51] Noh J., Kwon B., Han E., Park M., Yang W., Cho W., Yoo W., Khang G., Lee D. (2015). Amplification of oxidative stress by a dual stimuli-responsive hybrid drug enhances cancer cell death. Nat. Commun..

[52] Liubchenko G., Böning G., Zacherl M., Rumiantcev M., Unterrainer L.M., Gildehaus F.J., Brendel M., Resch S., Bartenstein P., Ziegler S.I. (2024). Image-based dosimetry for [^225^Ac]Ac-PSMA-I&T therapy and the effect of daughter-specific pharmacokinetics. Eur. J. Nucl. Med. Mol. Imaging.

[53] Barinka C., Novakova Z., Motlova L. (2017). X-Ray Structure of Human Glutamate Carboxypeptidase II (GCPII) in Complex with a Urea Based Inhibitor PSMA 1007.

[54] Olsson M.H.M., Søndergaard C.R., Rostkowski M., Jensen J.H. (2011). PROPKA3: Consistent Treatment of Internal and Surface Residues in Empirical pKa Predictions. J. Chem. Theory Comput..

[55] Jorgensen W.L., Maxwell D.S., Tirado-Rives J. (1996). Development and Testing of the OPLS All-Atom Force Field on Conformational Energetics and Properties of Organic Liquids. J. Am. Chem. Soc..

[56] Phillips J.C., Hardy D.J., Maia J.D., Stone J.E., Ribeiro J.V., Bernardi R.C., Buch R., Fiorin G., Hénin J., Jiang W. (2020). Scalable molecular dynamics on CPU and GPU architectures with NAMD. J. Chem. Phys..

[57] McNutt A.T., Francoeur P., Aggarwal R., Masuda T., Meli R., Ragoza M., Sunseri J., Koes D.R. (2021). GNINA 1.0: Molecular docking with deep learning. J. Cheminform..

[58] Barducci A., Bussi G., Parrinello M. (2008). Well-tempered metadynamics: A smoothly converging and tunable free-energy method. Phys. Rev. Lett..

[59] Tribello G.A., Bonomi M., Branduardi D., Camilloni C., Bussi G. (2014). PLUMED 2: New feathers for an old bird. Comput. Phys. Commun..

[60] Seoane D.C., De Saint-Hubert M., Ahenkorah S., Vargas C.S., Ooms M., Struelens L., Koole M. (2022). Gamma counting protocols for the accurate quantification of 225Ac and 213Bi without the need for a secular equilibrium between parent and gamma-emitting daughter. EJNMMI Radiopharm. Chem..

[61] Ocampo-García B., Cruz-Nova P., Jiménez-Mancilla N., Luna-Gutiérrez M., Oros-Pantoja R., Lara-Almazán N., Pérez-Velasco D., Santos-Cuevas C., Ferro-Flores G. (2023). 225Ac-iPSMA-RGD for alpha-therapy dual targeting of stromal/tumor cell PSMA and integrins. Int. J. Mol. Sci..

[62] Liolios C., Sachpekidis C., Schäfer M., Kopka K. (2019). Bispecific Radioligands targeting Prostate Specific Membrane Antigen (PSMA) and Gastrin Releasing Peptide receptors (GRPr) on the surface of prostate cancer cells. J. Label. Compd. Radiopharm..

[63] Eder M., Schäfer M., Bauder-Wüst U., Haberkorn U., Eisenhut M., Kopka K. (2014). Preclinical evaluation of a bispecific low-molecular heterodimer targeting both PSMA and GRPR for improved PET imaging and therapy of prostate cancer. Prostate.

[64] Brühlmann S.A., Walther M., Blei M.K., Mamat C., Kopka K., Freudenberg R., Kreller M. (2024). Scalability study on [133La] LaCl3 production with a focus on potential clinical applications. EJNMMI Radiopharm. Chem..

[65] Brühlmann S.A., Kreller M., Pietzsch H.-J., Kopka K., Mamat C., Walther M., Reissig F. (2022). Efficient production of the PET radionuclide ^133^La for theranostic purposes in targeted alpha therapy using the ^134^Ba (p, 2n) ^133^La reaction. Pharmaceuticals.

[66] Nelson B.J.B., Wilson J., Andersson J.D., Wuest F. (2023). Theranostic Imaging Surrogates for Targeted Alpha Therapy: Progress in Production, Purification, and Applications. Pharmaceuticals.

