# Supplementary material for: [225Ac]Ac-iPSMA Radiopharmaceuticals for Targeted Alpha Therapy: Design, Synthesis, and Preclinical Evaluation
Source: Int J Mol Sci. 2026 Aug 31;27(17):7785. doi: 10.3390/ijms27177785 (PMC13565901; doi:10.3390/ijms27177785)
Supplement: Supplementary file 1 [file ijms-27-07785-s001.zip › ijms-4506220-supplementary.pdf]

[[225]Ac]Ac-iPSMA Radiopharmaceuticals for Targeted Alpha Therapy: Design, Synthesis, Preclinical Evaluation

Supplementary Material

Table of Content

1. *in silico* supplemental analyses
2. Characterization Data
3. Characterization Spectra

1. ***in silico*** supplemental analyses
2. Figures were obtained in Schrödinger Maestro 2024-4 v14.2.118 (Schrödinger LLC, NY, USA).

2.1. Molecular dynamics simulations

S1

(a)

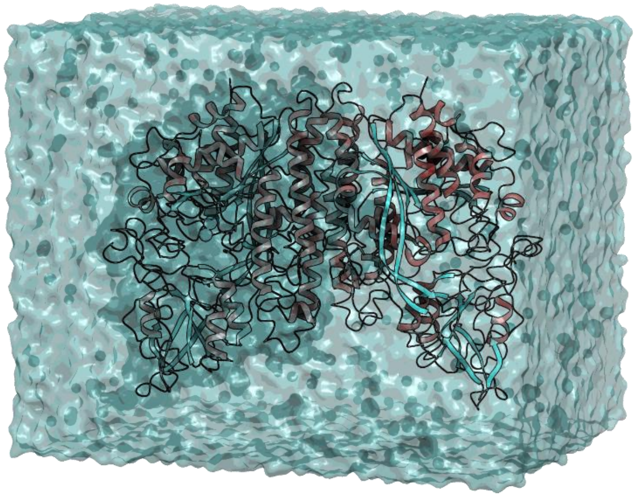

(b)

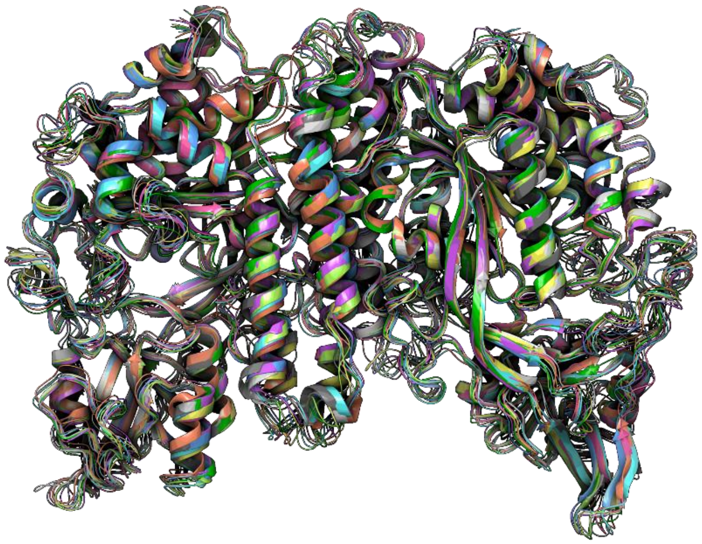

Figure S1. PSMA Molecular Dynamics simulation systems. (a) Full solvated MDS PSMA complex system. (b) Cluster of 10 different PSMA from MDS and RMSD evaluation. Protein is displayed as ribbon representation; ligand is displayed as ball-and-stick representation. PDB ID: 5O5T

(a)

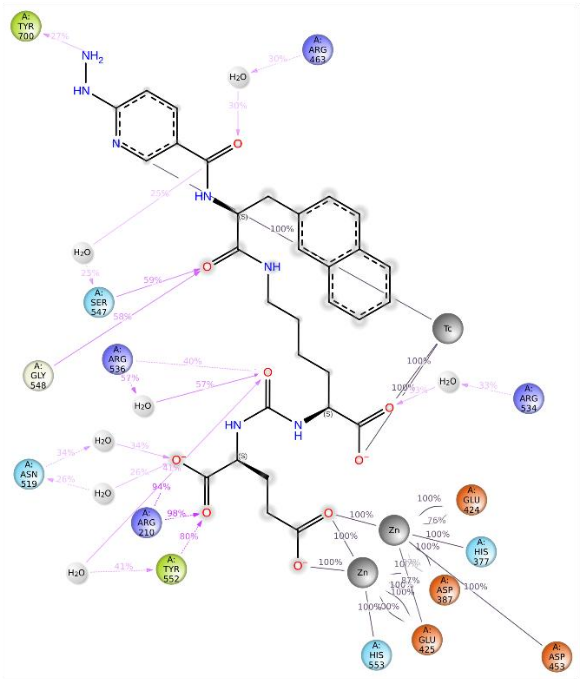

(b)

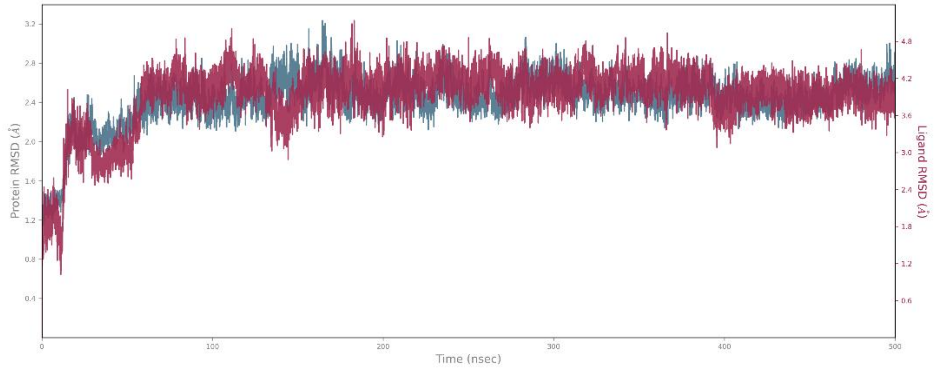

(c)

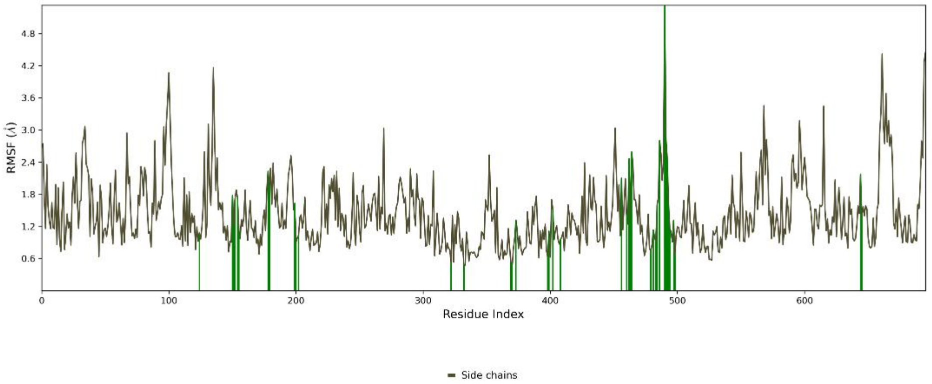

Figure S2. HYNIC-iPSMA Molecular Dynamics System. (a) 2D diagram of the H-iPSMA complex interactions (3 Å distance cutoff). (b) Ca RMS (root mean square) Deviation (RMSD) of PSMA and H-iPSMA during the Molecular Dynamics simulation. (c) Ca RMS Fluctuation (RMSF) of PSMA residues (green) and their side chains (gray).

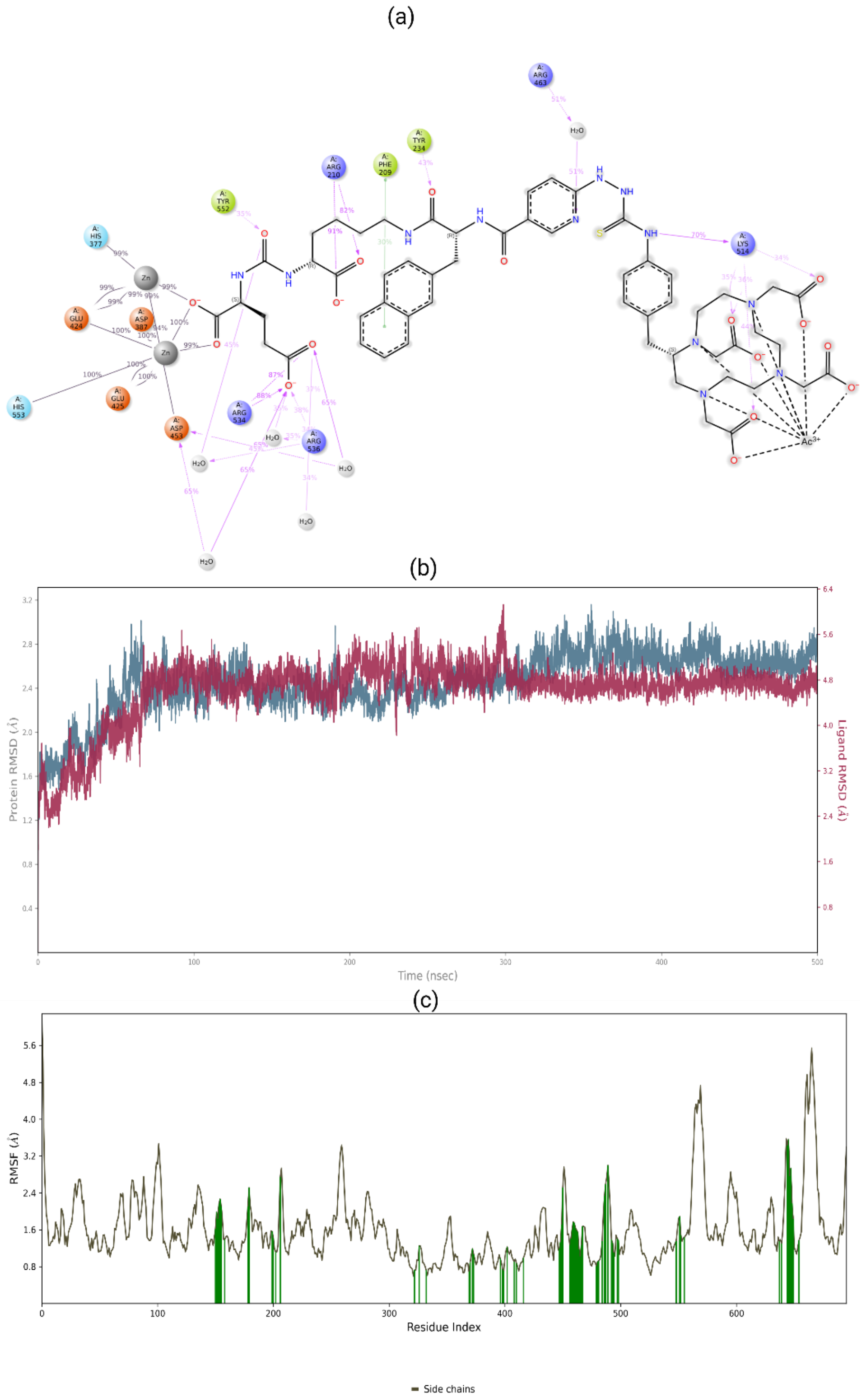

Figure S3. *p*-DOTA-NCS-Bn-iPSMA Molecular Dynamics System. (a) 2D diagram of the D-iPSMA complex interactions (3 Å distance cutoff). (b) Ca RMS (root mean square) Deviation (RMSD) of PSMA and D-iPSMA during the Molecular Dynamics simulation. (c) Ca RMS Fluctuation (RMSF) of PSMA residues (green) and their side chains (gray).

S4

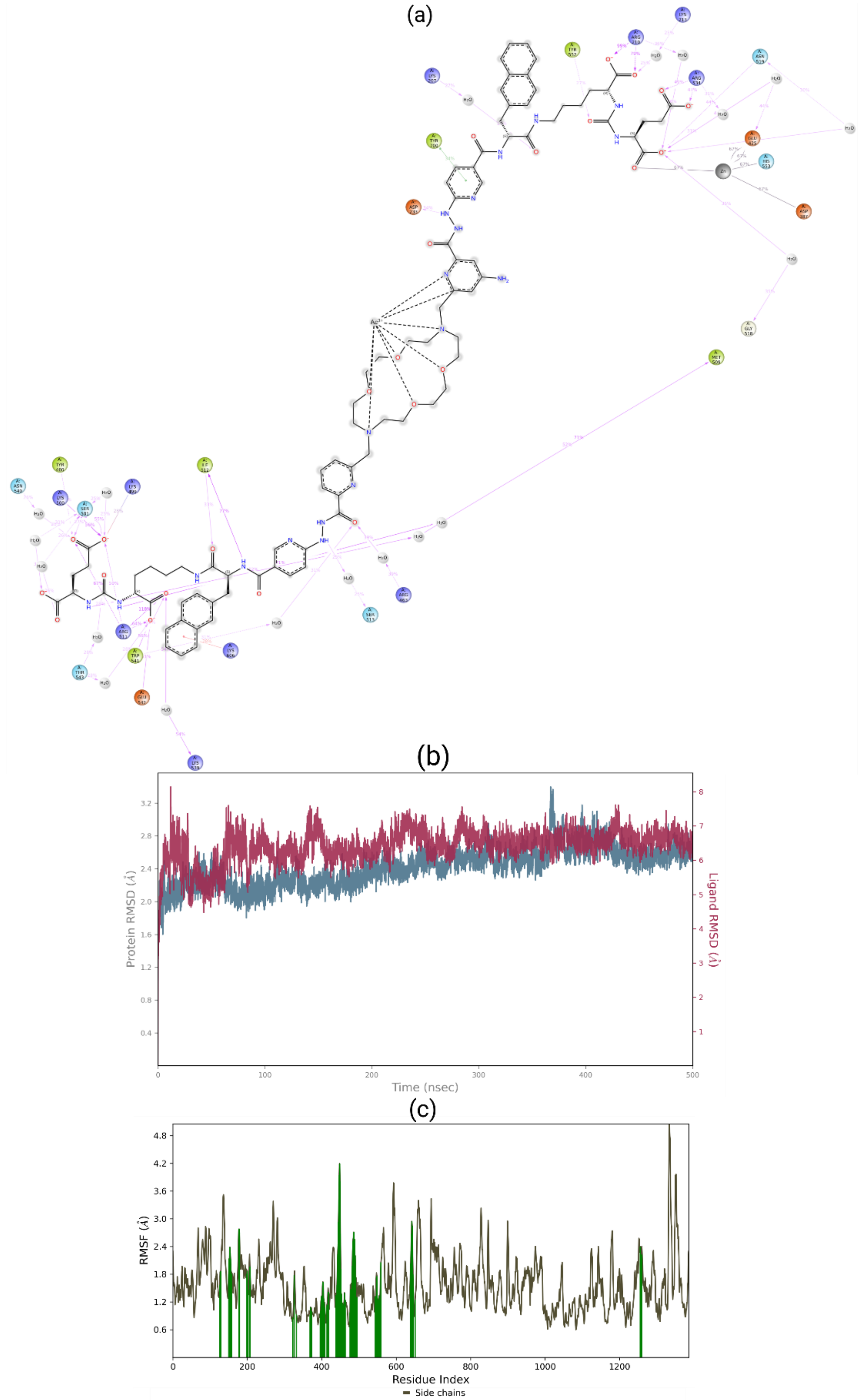

Figure S4. MACROPA-iPSMA Molecular Dynamics System. (a). 2D diagram of the M-iPSMA complex interactions (3 Å distance cutoff). (b) Ca RMS (root mean square) Deviation (RMSD) of PSMA and M-iPSMA during the Molecular Dynamics simulation. (c) Ca RMS Fluctuation (RMSF) of PSMA residues (green) and their side chains (gray).

3. Metadynamics FES plot

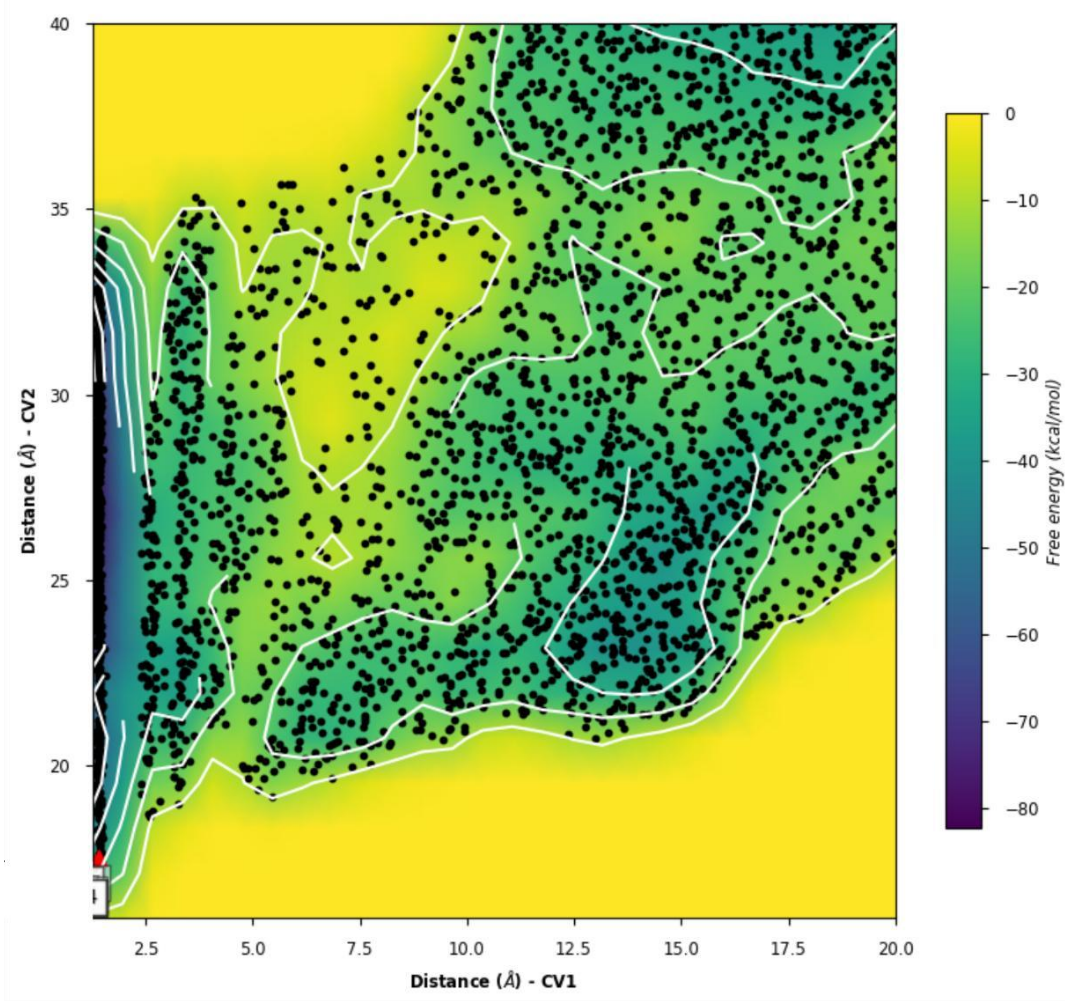

Figure S5. MACROPA-iPSMA Metadynamics Free Energy Surface plot.

2. Characterization Data

The signals under H_2O (~3.33 ppm) and DMSO-d6 (~2.50 ppm) remain partially masked; the shifts marked with "~" are approximate.

HYNIC-iPSMA.
^{1}H NMR (500 MHz, DMSO-d6). δ 9.50 (s, 1H), 8.68 (d, J = 10 Hz, 1H), 8.53 (d, J = 2.9 Hz, 1H), 8.15 (t, 1H), 8.03 (dd, J = 10, 3.5 Hz, 1H), 7.81 (m, 4H), 7.46 (m, 3H), 6.81 (d, J = 10 Hz, 1H), 6.31 (dd, 2H), 4.7 (m, 1H), 4.2–4.1 (m, 3H,), 3.06 (m, 4H), 2.28 (m, 3H), 1.92 (dq, 1H), 1.67 (m, 2H), 1.47 (dt, 1H), 1.31 (dt, 4H).

MACROPA-iPSMA.
FT-IR ν_{max} 1660 cm^{-1}. ^{1}H NMR (500 MHz, DMSO-d6). δ 10.8 (s, 1H), 10.3 (s, 1H), 9.8 (s, 1H), 9.2 (s, 1H), 9.1 (s, 1H), 8.50 (dd, 4H), 8.1 (m, 3H), 8.05 (d, J = 10 Hz, 1H), 7.92 (dd, J = 10, 5 Hz, 2H), 7.78 (m, 9H), 7.44 (m, 6H), 7.20 (d, 1H), 6.76 (s, 1H), 6.64 (m, 2H), 6.58 (d, 1H), 6.31 (dd, 4H), 4.74 (m, 5H), 4.51 (s, 2H), 4.07 (m, 5H), 3.84 (m, 8H), 3.23 (m, 4H), 3.11 (dd, 5H), 3.00 (q, 3H), 2.26 (m, 2H), 1.91 (dq, 3H), 1.67 (m, 5H), 1.48 (m, 3H), 1.35 (d, 5H), 1.26 (d, 4H). ESI-MS (m/z): Calc. 27728 [($C_{88}H_{107}N_{19}O_{24}$ + 2H$^+$) / 2], found 908.3.

***p*-NCS-Bn-iPSMA**
FT-IR ν_{max} 1350 cm^{-1}. ^{1}H NMR (500 MHz, DMSO-d6). δ 9.77 (s, 1H), 9.0 (s, 1H), 8.5 (m, 2H), 8.09 (m, 1H), 7.97 (m, 1H), 7.78 (m, 4H), 7.46 (m, 4H), 6.60 (dd, 1H), 6.32 (d, 2H), 4.7 (m, 1H), 4.05 (m, 2H), 3.55 (q, 5H), 3.08 (q, 6H), 2.25 (d, 2H), 1.89 (d, 2H), 1.71 (m, 1H), 1.61 (m, 1H), 1.49 (m, 1H), 1.35 (m, 2H). ESI-MS (m/z): Calc. 27728 [($C_{55}H_{70}N_{12}O_{17}S$ + H$^+$) / 2], found 602.34.

3 Characterization Spectra

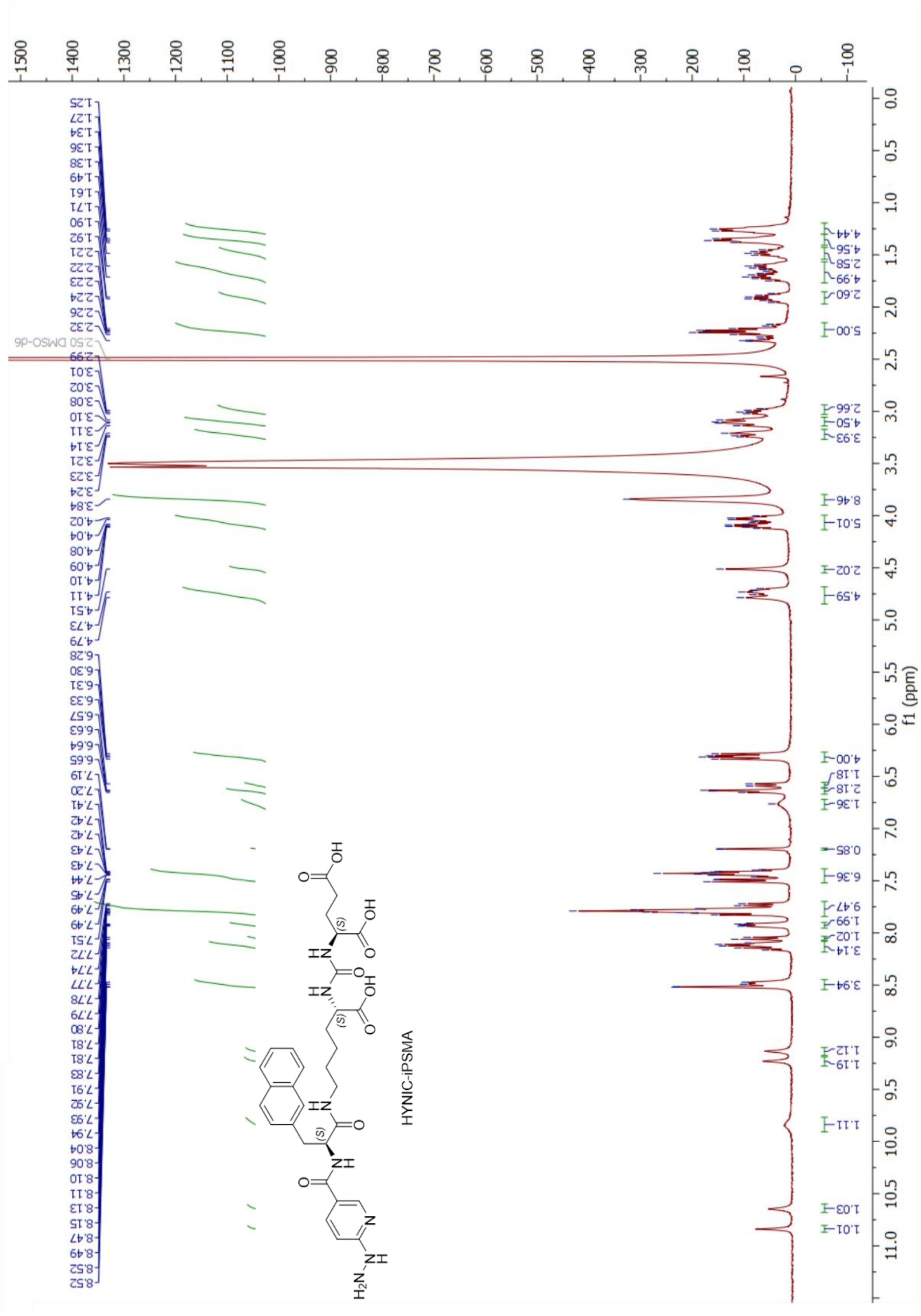

Figure S6. HYNIC-iPSMA. ^{1}H NMR. DMSO-d6. (500 MHz).

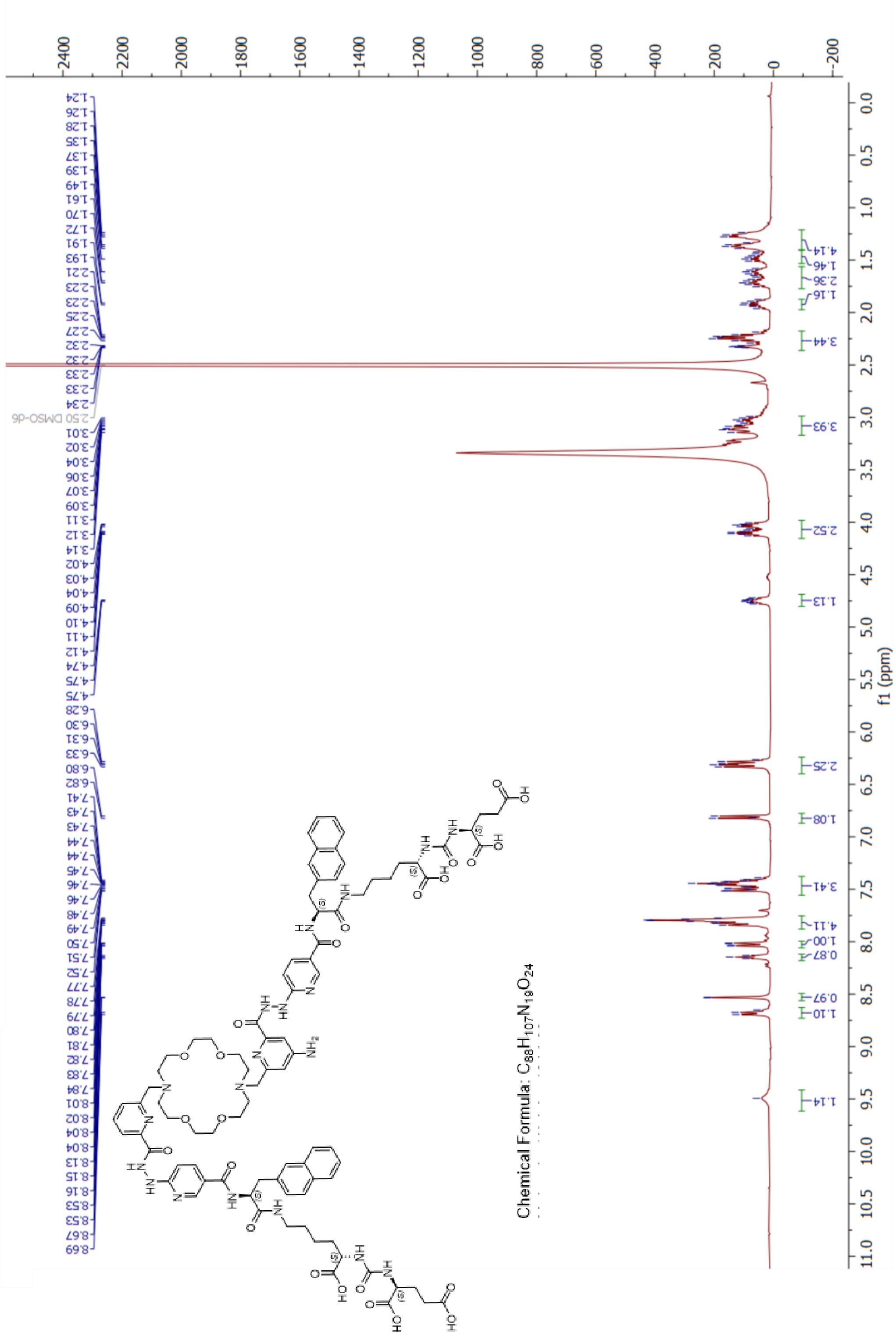

Figure S7. ^{1}H NMR. DMSO-d6. (500 MHz). MACROPA-iPSMA.

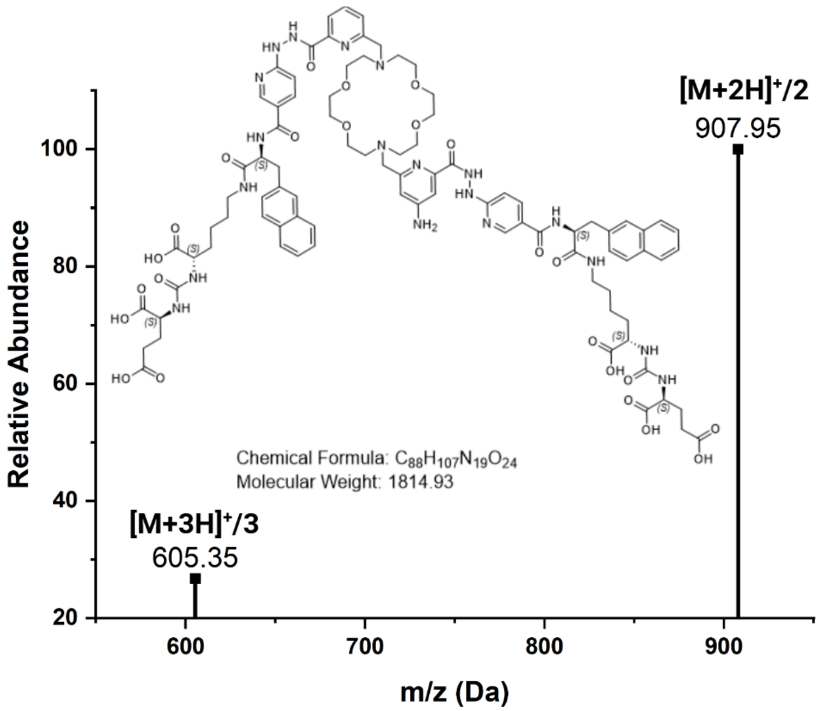

Figure S8. ESI-MS. MACROPA-iPSMA.

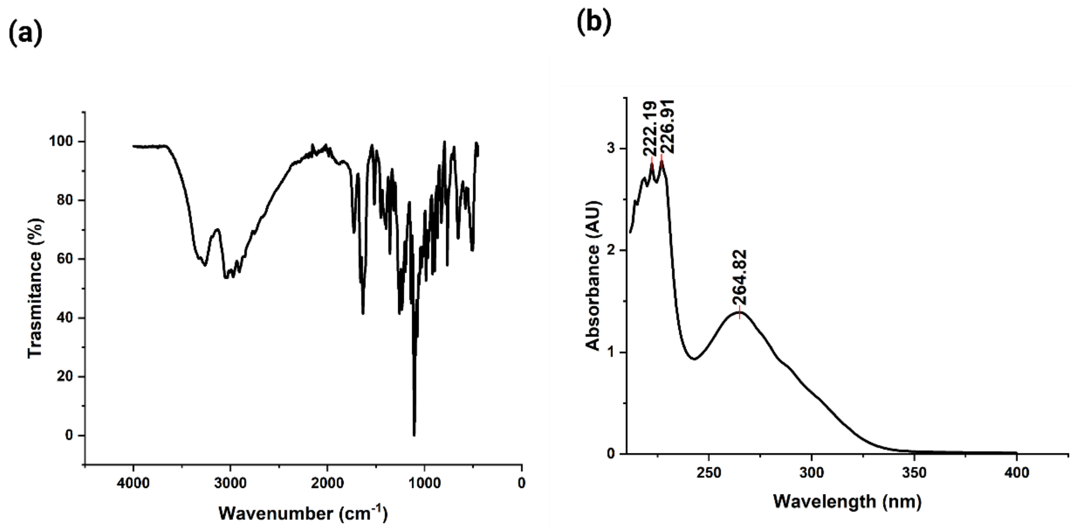

Figure S9. (a) FT-IR. (b) UV-Vis. MACROPA-iPSMA.

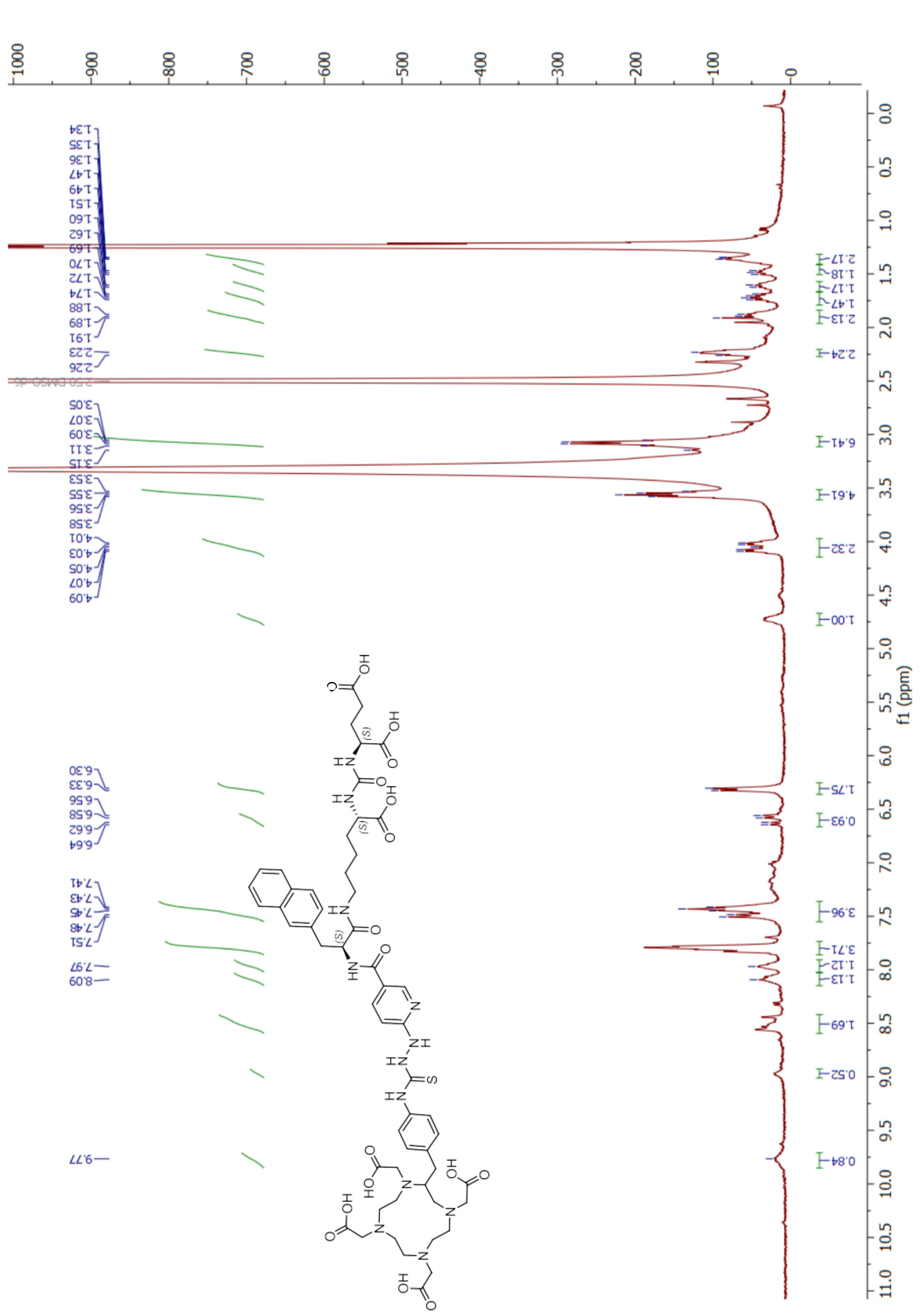

Figure S10. ^{1}H NMR. DMSO-d6. (500 MHz). *p*-NCS-DOTA-Bn-iPSMA

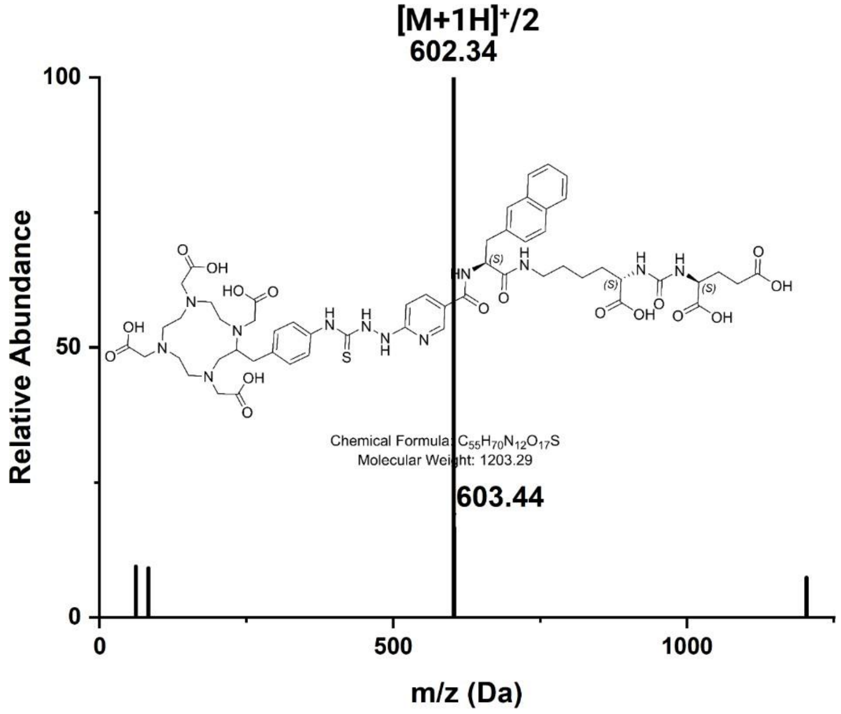

Figure S11. ESI-MS. *p*-NCS-DOTA-Bn-iPSMA

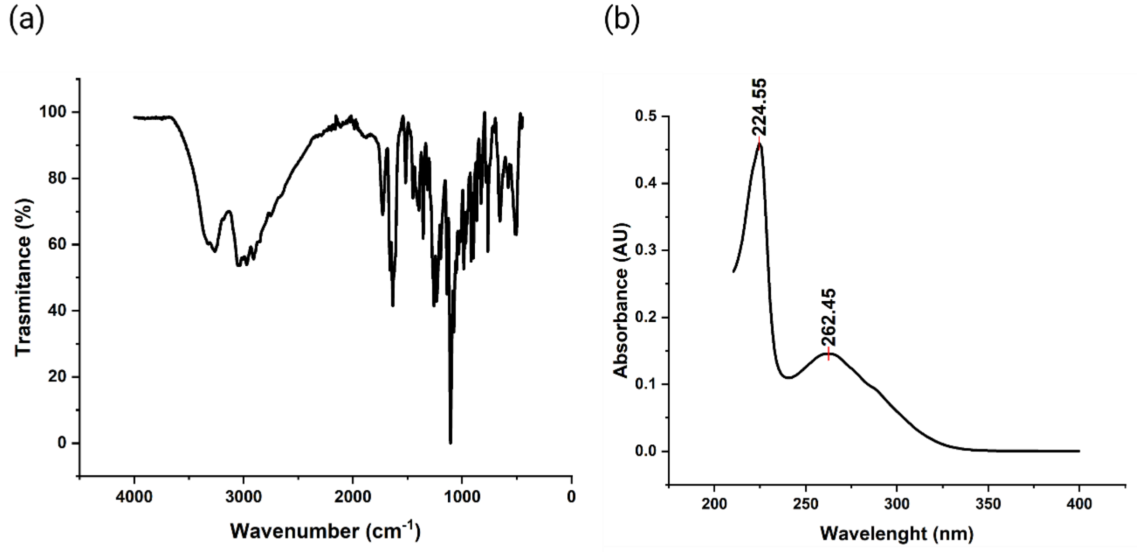

Figure S12. (a). FT-IR. (b). UV-Vis**.** *p*-NCS-DOTA-Bn-iPSMA

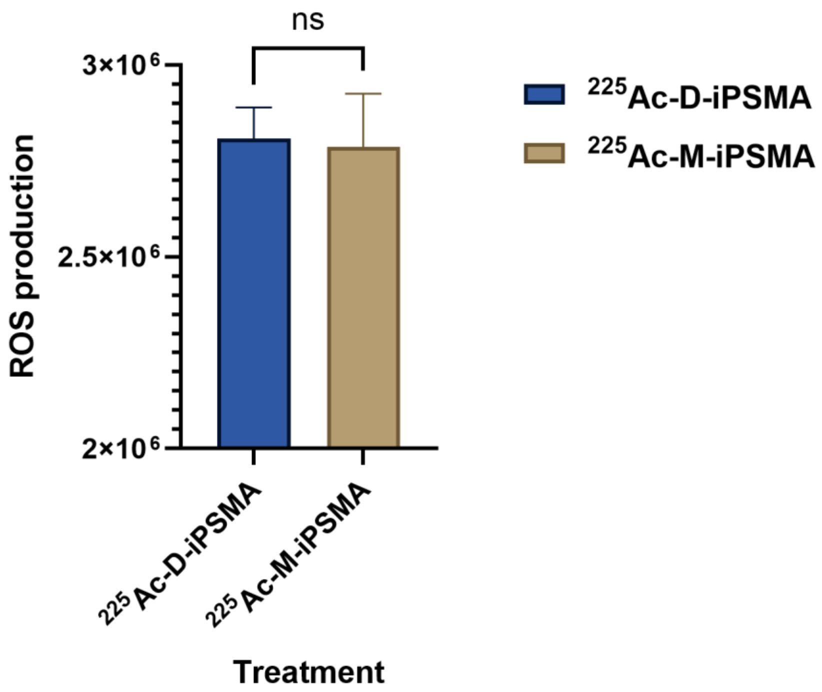

Figure S13. ROS production between [^{225}Ac]Ac-M-iPSMA and [^{225}Ac]Ac-D-iPSMA
